# Microscale whispering-gallery-mode light sources with lattice-confined atoms

**DOI:** 10.1038/s41598-021-93295-5

**Published:** 2021-07-06

**Authors:** Deshui Yu, Frank Vollmer

**Affiliations:** Living Systems Institute, Physics and Astronomy, University of Exeter, Exeter, EX4 4QD UK

**Keywords:** Quantum optics, Single photons and quantum effects

## Abstract

Microlasers, relying on the strong coupling between active particles and optical microcavity, exhibit fundamental differences from conventional lasers, such as multi-threshold/thresholdless behavior and nonclassical photon emission. As light sources, microlasers possess extensive applications in precision measurement, quantum information processing, and biochemical sensing. Here we propose a whispering-gallery-mode microlaser scheme, where ultracold alkaline-earth metal atoms, i.e., gain medium, are tightly confined in a two-color evanescent lattice that is in the ring shape and formed around a microsphere. To suppress the influence of the lattice-induced ac Stark shift on the moderately-narrow-linewidth laser transition, the red-detuned trapping beams operate at a magic wavelength while the wavelength of the blue-detuned trapping beam is set close to the other magic wavelength. The tiny mode volume and high quality factor of the microsphere ensure the strong atom-microcavity coupling in the bad-cavity regime. As a result, both saturation photon and critical atom numbers, which characterize the laser performance, are substantially reduced below unity. We explore the lasing action of the coupled system by using the Monte Carlo approach. Our scheme may be potentially generalized to the microlasers based on the forbidden clock transitions, holding the prospect for microscale active optical clocks in precision measurement and frequency metrology.

## Introduction

Microlasers, which are on the basis of intracavity particles strongly interacting with individual photons, possess unique properties, such as sub-millimeter dimension, multiple thresholds^1^/nonthreshold^2^, and nonclassical radiation^3^. Thus far, microlasers have been widely applied in photonic integrated circuits^4^, nanoplasmonics^5^, quantum information processing and computing^6^, and biochemical and optomechanical sensing^7–9^. The size of a particle-cavity coupled system is usually measured by two dimensionless parameters^3,10^, saturation photon number ${N}_{\mathrm{p}}^{\mathrm{s}}={\gamma }^{2}/4{g}^{2}$ and critical particle number ${N}_{\mathrm{a}}^{\mathrm{c}}=\kappa \gamma /4{g}^{2}$ with the loss rate *κ* of intracavity photons, the relaxation rate *γ* of the laser transition of active particles, and the coupling strength $g$ between one particle and one photon. When the number of intracavity photons exceeds ${N}_{\mathrm{p}}^{\mathrm{s}}$, the rate of stimulated emission from an atom surpasses the rate of spontaneous emission from this atom. By contrast, ${N}_{\mathrm{a}}^{\mathrm{c}}$ characterizes the half of the critical population inversion (i.e., laser threshold) for the laser activity. That is, the laser action occurs when the population inversion of active atoms is larger than $2{N}_{\mathrm{a}}^{\mathrm{c}}$. Indeed, ${N}_{\mathrm{a}}^{\mathrm{c}}$ is related to the so-called cooperativity parameter^10^ $\mathcal{C}=1/2{N}_{\mathrm{a}}^{\mathrm{c}}$. The conventional lasers operate with $\left({N}_{\mathrm{p}}^{\mathrm{s}},{N}_{\mathrm{a}}^{\mathrm{c}}\right)\gg 1$, thereby requiring a large number of particles and photons to sustain the laser oscillation. By contrast, the size of a laser system is significantly suppressed in the strong-coupling limit, $\left({N}_{\mathrm{p}}^{\mathrm{s}},{N}_{\mathrm{a}}^{\mathrm{c}}\right)\ll 1$, where even one photon can saturate the particle’s transition and even one particle can strongly affect the intracavity field. Consequently, interesting quantum effects, such as single-photon emission and thresholdless lasing^3^, are exhibited.

Suppressing $\left({N}_{\mathrm{p}}^{\mathrm{s}},{N}_{\mathrm{a}}^{\mathrm{c}}\right)$ relies on a large particle-cavity coupling strength $g$ that is proportional to $\sqrt{Q/{V}_{\text{eff}}}$ with the quality factor $Q$ and effective mode volume *V*_eff_ of the cavity^3,10^. Thus far, various particle-cavity structures have accessed the strong-coupling regime, including one atom/ion/molecule/quantum dot situated in an optical cavity^3,11–13^, a superconducting qubit interacting with an *LC* resonator via the electric/magnetic field (i.e., circuit QED^14^), and single molecule placed inside a plasmonic nanocavity at room temperature^15^. In comparison, the whispering-gallery-mode (WGM) microcavities, which confine the optical waves by means of the total internal reflection, own the features of ultrahigh $Q$ factor (~ 10^9^)^16,17^ and tiny mode volume (a few hundreds of µm^3^ measured according to the maximum light intensity inside the microcavity^18^). Thus, the WGM microcavities are an excellent platform for implementing microlasers^19,20^.

Recently, the strong coupling between cold caesium atoms and a toroidal microcavity has been demonstrated in^21,22^. However, the short transient interaction time (~ µs) seriously restricts the implementation of a microlaser. To this end, confining active particles in an optical lattice potential that is formed inside or in the vicinity of a microcavity is an efficient solution^23,24^. The particle-cavity interaction time may be extended over 10^2^ s^25^. In addition, the particles moving in the so-called Lamb–Dicke regime, where the ground-vibrational-state size of a particle in a potential trap is much smaller than the radiation wavelength of the particle, allows a Doppler-free as well as recoil-free spectroscopy^26^. Further, when the trapping laser beams operate at the so-called magic wavelengths, two laser-transition states of the particles have the same dynamical polarizability and experience the same trap-induced ac Stark shift^27^. This is of particular importance to the application in the field of time and frequency metrology. A few methods have been proposed for trapping neutral atoms around WGM microcavities, such as the three-level atoms interacting with two WGMs of a microcavity^28,29^ and the two-color evanescent trap formed around a microcavity^30^. However, none of them operate at the magic-wavelength condition.

In this work, we propose a WGM microlaser scheme with a small number of neutral ^88^Sr atoms strongly interacting with a high-$Q$ microsphere. The (5*s*^2^)^1^*S*_0_–(5*s*5*p*)^3^*P*_1_ (*m* = 0) intercombination transition is chosen as the laser transition. Ultracold atoms are trapped in a ring-shaped evanescent lattice that is formed around the microsphere. The trapping-beam wavelengths include one red-detuned magic wavelength and one blue-detuned wavelength that is close to the other magic wavelength. The two-color lattice potential contains tens of sites with each site depth reaching tens of µK. The effective WGM volume *V*_eff_ is about $827$ µm^3^, leading to an atom-microcavity coupling strength as high as $g\sim 2\mathrm{\pi }\times 2.8$ MHz. The resultant saturation photon ${N}_{\mathrm{p}}^{\mathrm{s}}$ and critical particle ${N}_{\mathrm{a}}^{\mathrm{c}}$ numbers are well below unity. The properties of the microlasing action with a few atoms interacting with the microcavity is studied through the Monte Carlo wave-function method. Our microlaser scheme paves the way towards micro-sized light sources for photonic integrated circuits and sensing applications.

## Results

### Physical model

**Figure 1:**
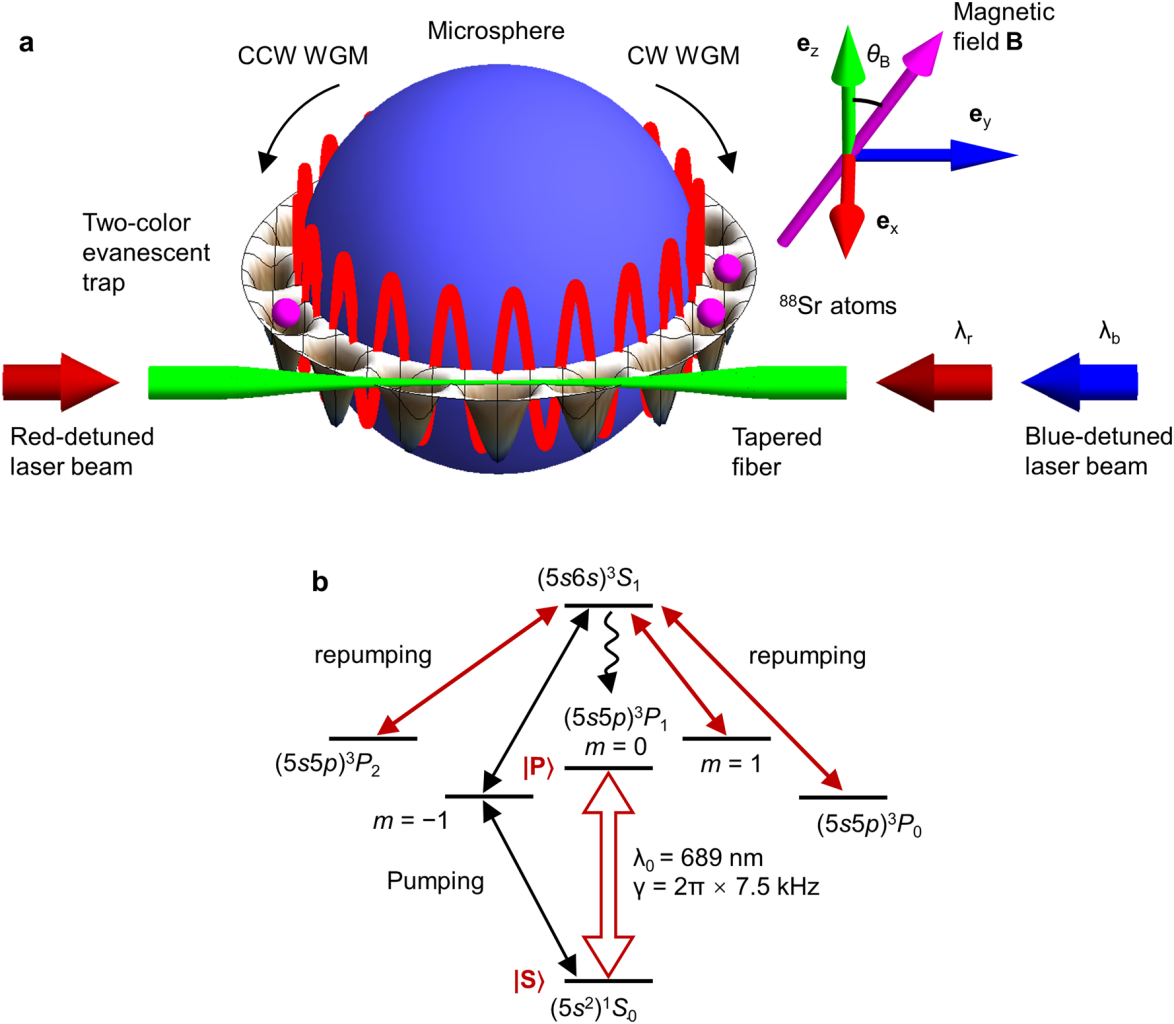
General scheme of atom-microcavity coupling. (**a**) Neutral ^88^Sr atoms are confined around a microsphere (radius $R=5.03$ µm) by a two-color (blue-detuned wavelength ${\lambda }_{\mathrm{b}}=392.58$ nm and red-detuned magic wavelength ${\lambda }_{\mathrm{r}}=832.69$ nm) evanescent lattice that is in the ring shape. All trapping beams are coupled into the microsphere via a tapered fiber. The quantum axis is defined by a constant magnetic field **B** that is in the $y-z$ plane. The angle between the ${\text{e}}_{\mathrm{z}}$ axis and **B** is *θ*_B_. The atoms interact with a pair of degenerate TE-polarized $\left({n}_{0}=1,{l}_{0}=60,\pm {m}_{0}\right)$ WGMs with ${m}_{0}={l}_{0}$. Two WGMs are resonant to the intercombination transition of ^88^Sr. (**b**) Level structure of ^88^Sr. The wavelength and natural linewidth of the laser $\left| S \right\rangle$−$\left| P \right\rangle$ transition with $\left| S \right\rangle$ ≡ (5*s*^2^)^1^*S*_0_ and $\left| P \right\rangle$ ≡ (5*s*5*p*)^3^*P*_1_ (*m* = 0) are ${\lambda }_{0}=689$ nm and $\gamma =2\mathrm{\pi }\times 7.5$ kHz, respectively. The pump lights are resonantly coupled with the (5*s*^2^)^1^*S*_0_–(5*s*5*p*)^3^*P*_1_ (*m* =  − 1) and (5*s*5*p*)^3^*P*_1_ (*m* =  − 1)–(5*s*6*s*)^3^*S*_1_ transitions. The repump beams resonantly derive the (5*s*5*p*)^3^*P*_0,2_–(5*s*6*s*)^3^*S*_1_ and (5*s*5*p*)^3^*P*_1_ (*m* = 1)–(5*s*6*s*)^3^*S*_1_ transitions. The atoms accumulate in $\left| P \right\rangle$ through the spontaneous emission (represented by the wavy curve) from (5*s*6*s*)^3^*S*_1_ to $\left| P \right\rangle$.

Figure 1a illustrates the schematic diagram of the atom-microcavity coupled system under study. An ensemble of neutral ^88^Sr atoms is confined in a ring-shaped optical lattice (in the $x-y$ plane) that is formed around a silica microsphere (radius *R*). An external magnetic field **B** (in the $y-z$ plane) is applied to define the quantization axis. The angle of **B** with respect to the ${\mathbf{e}}_{\mathrm{z}}$ axis is *θ*_B_. A pair of TE-polarized clockwise (CW) and counterclockwise (CCW) WGMs are resonantly coupled to the $\left| S \right\rangle$−$\left| P \right\rangle$ intercombination transition of ^88^Sr via the evanescent field. Here, we have defined $\left| S \right\rangle$≡ (5*s*^2^)^1^*S*_0_ and $\left| P \right\rangle$ ≡ (5*s*5*p*)^3^*P*_1_ (*m* = 0). The transition wavelength in free space is ${\lambda }_{0}=689.45$ nm with a moderately narrow linewidth $\gamma =2\mathrm{\pi }\times 7.5$ kHz (Fig. 1b). The tiny mode volume of the microsphere allows the access to the strong-coupling regime and further the implementation of a microlaser.

The ring-shaped optical lattice is created by combining the evanescent fields of a blue-detuned travelling WGM and a red-detuned standing-wave WGM in microsphere. Both WGMs are TE-polarized and far-off-resonance with respect to *λ*_0_, thereby suppressing the inelastic photon scattering by atoms. All trapping beams are linearly polarized along the *z*-direction and coupled into the microsphere through a tapered fiber that is aligned along the *y*-direction (Fig. 1a). The two-color evanescent trapping was firstly proposed and demonstrated in optical nanofibers and waveguides^31–34^, where the blue-detuned (red-detuned) optical potential pushes (attracts) atoms away from (towards) the dielectric surface. Here, we extend the relevant application to the WGM microsphere, where, in addition, two-color trapping beams operate at or close to the so-called magic wavelengths so that two atomic states $\left| S \right\rangle$ and $\left| P \right\rangle$ approximately experience the same ac Stark shift^27^.

We first consider choosing the microsphere’s radius *R*. A TE-polarized WGM in microsphere is characterized by a set of integers $\left(n\ge 1,l\ge 0,m\right)$ with $-l\le m\le l$. Actually, $\left(n,l,m\right)$ denote $n$, $\left(l-\left|m\right|+1\right)$ and $2\left|m\right|$ intensity maxima of the WGM along the radial, polar and azimuthal directions, respectively. A WGM with a small $l$ enhances the evanescent field, facilitating a deep-enough optical potential and the strong atom-microcavity coupling^19^. For a certain $l$, the microsphere’s radius *R* should be determined by the atomic transition frequency ${\omega }_{0}=2\mathrm{\pi }c/{\lambda }_{0}$. The refractive index of silica at a light wavelength *λ* can be evaluated by using the Sellmeier formula for the relative permittivity.

1 $$\epsilon \left(\lambda \right)=1+\frac{0.6961663\times {\lambda }^{2}}{{\lambda }^{2}-{0.0684043}^{2}}+\frac{0.4079426\times {\lambda }^{2}}{{\lambda }^{2}-{0.1162414}^{2}}+\frac{0.8974794\times {\lambda }^{2}}{{\lambda }^{2}-{9.896161}^{2}}.$$

In above equation, *λ* is in units of µm. According to the explicit asymptotic formula of the resonance frequency of a $\left(n,l,m\right)$ WGM derived in^35^, we set *R* = 5.03 µm and the intercombination transition of ^88^Sr resonantly interacts with the fundamental CW $\left({n}_{0}=1,{l}_{0}=60,{m}_{0}\right)$ and CCW $\left({n}_{0}=1,{l}_{0}=60,-{m}_{0}\right)$ WGMs with ${m}_{0}={l}_{0}$. In what follows, we name $\left({n}_{0},{l}_{0},\pm {m}_{0}\right)$ the lasing modes.

Before computing the optical potential, we first consider the blue-detuned travelling ${\mathbf{E}}_{\mathrm{b}}$ and red-detuned standing-wave ${\mathbf{E}}_{\mathrm{r}}$ WGMs that are used to trap ^88^Sr atoms. The blue-detuned trapping beam (${\mathbf{e}}_{\mathrm{z}}$-polarization, wavelength in free space ${\lambda }_{\mathrm{b}}<{\lambda }_{0}$ and wavenumber ${k}_{\mathrm{b}}=2\mathrm{\pi }/{\lambda }_{\mathrm{b}}$) is coupled into the fundamental TE-polarized WGM $\left({n}_{\mathrm{b}},{l}_{\mathrm{b}},{m}_{\mathrm{b}}={l}_{\mathrm{b}}\right)$ in microsphere via the tapered fiber (Fig. 1a). In spherical coordinates $\left(\rho ,\theta ,\varphi \right)$, the amplitude vector of the blue-detuned WGM consists of the intracavity field (inside the microsphere)^36^.

2 $${\mathbf{E}}_{\mathrm{b}}\left(\rho <R,\theta ,\varphi \right)={E}_{\mathrm{i},\mathrm{b}}\frac{{\psi }_{{l}_{\mathrm{b}}}\left(\sqrt{\epsilon \left({\lambda }_{\mathrm{b}}\right)}{k}_{\mathrm{b}}\rho \right)}{\sqrt{\epsilon \left({\lambda }_{\mathrm{b}}\right)}{k}_{\mathrm{b}}\rho }{\text{X}}_{{l}_{\mathrm{b}},{m}_{\mathrm{b}}}\left(\theta ,\varphi \right),$$

and the evanescent field (outside the microsphere).

3 $${\mathbf{E}}_{\mathrm{b}}\left(\rho >R,\theta ,\varphi \right)={E}_{\mathrm{o},\mathrm{b}}\frac{{\xi }_{{l}_{\mathrm{b}}}\left({k}_{\mathrm{b}}\rho \right)}{{k}_{\mathrm{b}}\rho }{\text{X}}_{{l}_{\mathrm{b}},{m}_{\mathrm{b}}}\left(\theta ,\varphi \right),$$

with the Riccati–Bessel functions ${\psi }_{l}\left(\zeta \right)=\zeta {j}_{l}\left(\zeta \right)$, ${\xi }_{l}\left(\zeta \right)={\psi }_{l}\left(\zeta \right)-i{\chi }_{l}\left(\zeta \right)$, and ${\chi }_{l}\left(\zeta \right)=-\zeta {n}_{l}\left(\zeta \right)$. Here, ${j}_{l}\left(\zeta \right)$ and ${n}_{l}\left(\zeta \right)$ are the spherical Bessel functions of the first and second kind, respectively, of the order $l$. The vector harmonics is defined as ${\text{X}}_{l,m}\left(\theta ,\varphi \right)=\left[\nabla {Y}_{l,m}\left(\theta ,\varphi \right)\right]\times \mathbf{r}$ with the spherical harmonics ${Y}_{l,m}\left(\theta ,\varphi \right)$. The boundary conditions at the microsphere’s surface give the relation between amplitudes *E*_i,b_ and *E*_o,b_

4 $${E}_{\mathrm{i},\mathrm{b}}\frac{{\psi }_{{l}_{\mathrm{b}}}\left(\sqrt{\epsilon \left({\lambda }_{\mathrm{b}}\right)}{k}_{\mathrm{b}}R\right)}{\sqrt{\epsilon \left({\lambda }_{\mathrm{b}}\right)}{k}_{\mathrm{b}}R}={E}_{\mathrm{o},\mathrm{b}}\frac{{\xi }_{{l}_{\mathrm{b}}}\left({k}_{\mathrm{b}}R\right)}{{k}_{\mathrm{b}}R},$$

and the modal equation for the resonance wavelength *λ*_b_.

5 $$\sqrt{\epsilon \left({\lambda }_{\mathrm{b}}\right)}\frac{{\psi }_{{l}_{\mathrm{b}}}^{\prime}\left(\sqrt{\epsilon \left({\lambda }_{\mathrm{b}}\right)}{k}_{\mathrm{b}}R\right)}{{\psi }_{{l}_{\mathrm{b}}}\left(\sqrt{\epsilon \left({\lambda }_{\mathrm{b}}\right)}{k}_{\mathrm{b}}R\right)}=\frac{{\xi }_{{l}_{\mathrm{b}}}^{\prime}\left({k}_{\mathrm{b}}R\right)}{{\xi }_{{l}_{\mathrm{b}}}\left({k}_{\mathrm{b}}R\right)}.$$

By contrast, two counter-propagating red-detuned laser beams (${\mathbf{e}}_{\mathrm{z}}$-polarization, wavelength in free space ${\lambda }_{\mathrm{r}}>{\lambda }_{0}$ and wavenumber ${k}_{\mathrm{r}}=2\mathrm{\pi }/{\lambda }_{\mathrm{r}}$) are respectively coupled into the degenerate TE-polarized $\left({n}_{\mathrm{r}},{l}_{\mathrm{r}},\pm {m}_{\mathrm{r}}\right)$ WGMs with ${m}_{\mathrm{r}}={l}_{\mathrm{r}}-2$, forming a ring-shaped standing wave in microsphere. Here, we do not choose the fundamental WGMs to form the standing wave for the reason of creating a closed three-dimensional optical trap. The amplitude vector of the standing-wave WGM contains the intracavity component.

6 $${\mathbf{E}}_{\mathrm{r}}\left(\rho <R,\theta ,\varphi \right)={E}_{\mathrm{i},\mathrm{r}}\frac{{\psi }_{{l}_{\mathrm{r}}}\left(\sqrt{\epsilon \left({\lambda }_{\mathrm{r}}\right)}{k}_{\mathrm{r}}\rho \right)}{\sqrt{\epsilon \left({\lambda }_{\mathrm{r}}\right)}{k}_{\mathrm{r}}\rho }\left[{\text{X}}_{{l}_{\mathrm{r}},{m}_{\mathrm{r}}}\left(\theta ,\varphi \right)+{\text{X}}_{{l}_{\mathrm{r}},-{m}_{\mathrm{r}}}\left(\theta ,\varphi \right)\right],$$

and the evanescent component.

7 $${\mathbf{E}}_{\mathrm{r}}\left(\rho >R,\theta ,\varphi \right)={E}_{\mathrm{o},\mathrm{r}}\frac{{\xi }_{{l}_{\mathrm{r}}}\left({k}_{\mathrm{r}}\rho \right)}{{k}_{\mathrm{r}}\rho }\left[{\text{X}}_{{l}_{\mathrm{r}},{m}_{\mathrm{r}}}\left(\theta ,\varphi \right)+{\text{X}}_{{l}_{\mathrm{r}},-{m}_{\mathrm{r}}}\left(\theta ,\varphi \right)\right].$$

According to the boundary conditions at the microsphere’s surface, one has the relation.

8 $${E}_{\mathrm{i},\mathrm{r}}\frac{{\psi }_{{l}_{\mathrm{r}}}\left(\sqrt{\epsilon \left({\lambda }_{\mathrm{r}}\right)}{k}_{\mathrm{r}}R\right)}{\sqrt{\epsilon \left({\lambda }_{\mathrm{r}}\right)}{k}_{\mathrm{r}}R}={E}_{\mathrm{o},\mathrm{r}}\frac{{\xi }_{{l}_{\mathrm{r}}}\left({k}_{\mathrm{r}}R\right)}{{k}_{\mathrm{r}}R},$$

and the modal equation for the resonance wavelength *λ*_r_.

9 $$\sqrt{\epsilon \left({\lambda }_{\mathrm{r}}\right)}\frac{{\psi }_{{l}_{\mathrm{r}}}^{\prime}\left(\sqrt{\epsilon \left({\lambda }_{\mathrm{r}}\right)}{k}_{\mathrm{r}}R\right)}{{\psi }_{{l}_{\mathrm{r}}}\left(\sqrt{\epsilon \left({\lambda }_{\mathrm{r}}\right)}{k}_{\mathrm{r}}R\right)}=\frac{{\xi }_{{l}_{\mathrm{r}}}^{\prime}\left({k}_{\mathrm{r}}R\right)}{{\xi }_{{l}_{\mathrm{r}}}\left({k}_{\mathrm{r}}R\right)}.$$

For the convenience of analysis, we further simplify the expression of the vector spherical harmonics ${\text{X}}_{l,m}\left(\theta ,\varphi \right)$. The polarizations of two-color trapping WGMs are mainly along the polar direction ${\mathbf{e}}_{\theta }$ when $R\gg {\lambda }_{u=\mathrm{b},\mathrm{r}}$. In addition, the intracavity light fields are tightly confined in the vicinity of the equator of the microsphere, i.e., $\theta \sim \mathrm{\pi }/2+\delta \theta$. Thus, the harmonic vector ${\text{X}}_{l,m}\left(\theta ,\varphi \right)$ with $l={l}_{u=\mathrm{b},\mathrm{r}}$ and $m={m}_{u=\mathrm{b},\mathrm{r}}$ is reduced to.

10 $${\text{X}}_{l,m}\left(\theta ,\varphi \right)\sim {\mathrm{\Theta }}_{l,m}\left(\frac{\pi }{2}+\delta \theta \right){\mathbf{e}}_{\mathrm{z}}=\frac{{\left(\left|m\right|/\pi \right)}^{1/4}}{\sqrt{{2}^{l-\left|m\right|}\left(l-\left|m\right|\right)!}}{H}_{l-\left|m\right|}\left(\sqrt{\left|m\right|}\delta \theta \right){e}^{-\left|m\right|{\left(\delta \theta \right)}^{2}/2}{e}^{im\varphi }{\mathbf{e}}_{\mathrm{z}},$$

with the Hermite polynomials ${H}_{q}\left(\zeta \right)$ of the degree *q*, and thus, the evanescent fields ${\mathbf{E}}_{u=\mathrm{b},\mathrm{r}}\left(\rho >R,\theta ,\varphi \right)$ are approximately polarized in the ${\mathbf{e}}_{\mathrm{z}}$ axis.

After obtaining ${\mathbf{E}}_{\mathrm{b}}$ and ${\mathbf{E}}_{\mathrm{r}}$, we consider the optical potential for trapping ^88^Sr atoms. The optical potentials produced by far-off-resonance blue- and red-detuned evanescent fields take the form.

11 $${U}_{u=\mathrm{b},\mathrm{r}}^{\left(j=\mathrm{S},\mathrm{P}\right)}\left(\rho >R,\theta ,\varphi \right)=-\frac{{\alpha }_{j}\left({\lambda }_{u},{\theta }_{\text{B}}\right)}{4}{\left|{\mathbf{E}}_{u}\left(\rho >R,\theta ,\varphi \right)\right|}^{2},$$

where ${\alpha }_{j=\mathrm{S},\mathrm{P}}$ denotes the dynamic polarizability of the atom in $\left| S \right\rangle$ and $\left| P \right\rangle$, respectively. The polarizability ${\alpha }_{j=\mathrm{S},\mathrm{P}}$ depends on the trapping beam wavelength ${\lambda }_{u=\mathrm{b},\mathrm{r}}$ and the quantization direction *θ*_B_ and can be computed by using the approach in^37^ and the data listed in^38^ (see “Methods”). As pointed out in^27^, setting ${\lambda }_{u=\mathrm{b},\mathrm{r}}$ at the so-called magic wavelengths minimizes the trapping-beam-induced ac Stark shift of the $\left| S \right\rangle$ −$\left| P \right\rangle$ intercombination line and also maximizes the spatial overlap between external motions of the atom in $\left| S \right\rangle$ and $\left| P \right\rangle$. However, it is challenging to find a pair of $\left({n}_{\mathrm{b}},{l}_{\mathrm{b}},{m}_{\mathrm{b}}\right)$ and $\left({n}_{\mathrm{r}},{l}_{\mathrm{r}},\pm {m}_{\mathrm{r}}\right)$ WGMs that simultaneously satisfy the magic-wavelength conditions for a given radius *R* (determined by the lasing WGMs) of the microsphere. In this case, we choose the red-detuned standing-wave WGM operating at the magic wavelength while the wavelength of the blue-detuned travelling WGM is set close to the other magic wavelength.

**Figure 2:**
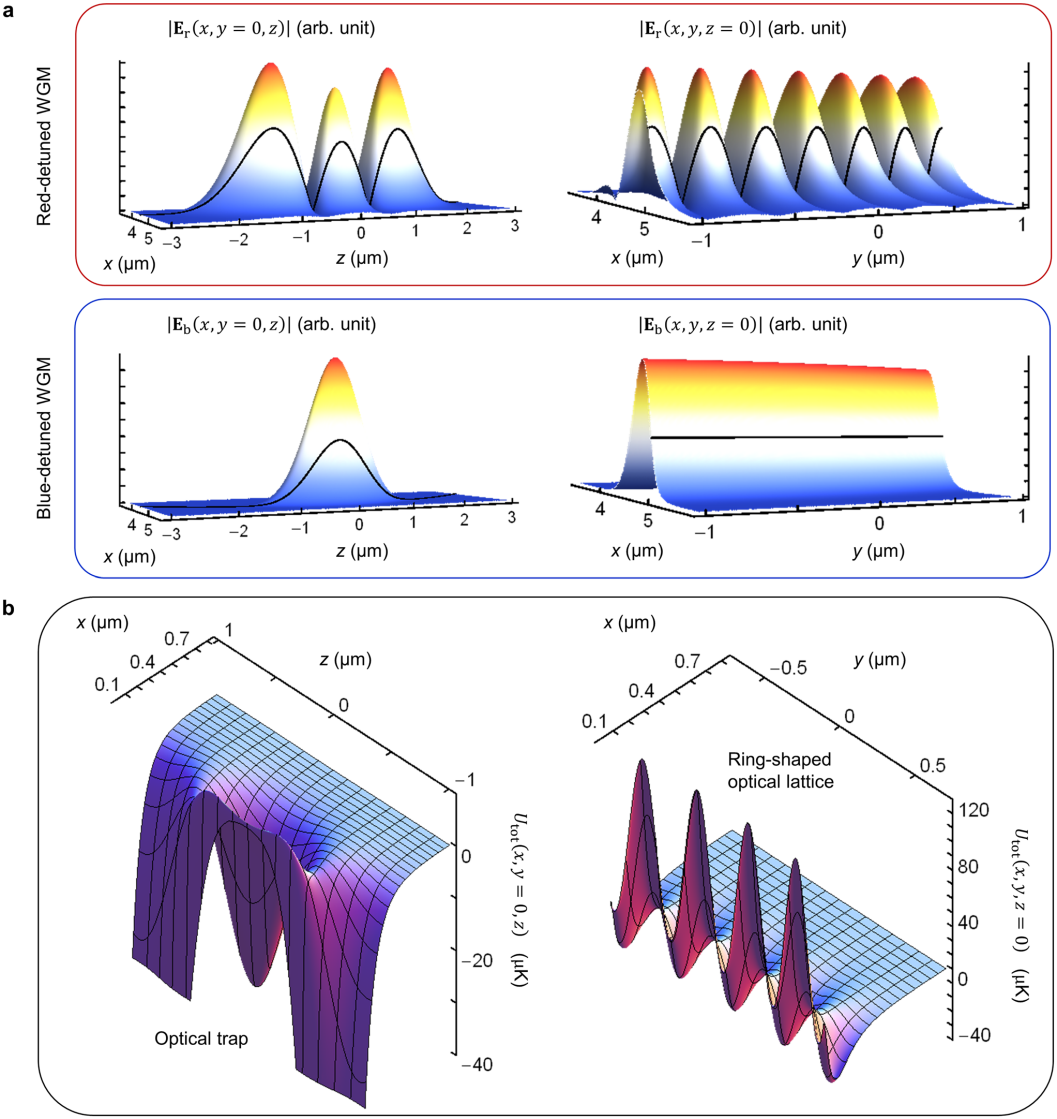
Ring-shaped optical lattice. (**a**) Electric-field distributions of the red-detuned standing-wave $\left({n}_{\mathrm{r}}=1,{l}_{\mathrm{r}}=49,{m}_{\mathrm{r}}=47\right)+\left({n}_{\mathrm{r}}=1,{l}_{\mathrm{r}}=49,-{m}_{\mathrm{r}}\right)$ WGM at the magic wavelength ${\lambda }_{\mathrm{r}}=832.69$ nm and the blue-detuned travelling $\left({n}_{\mathrm{b}}=1,{l}_{\mathrm{b}}=109,{m}_{\mathrm{b}}=109\right)$ WGM at the wavelength ${\lambda }_{\mathrm{b}}=392.58$ nm. Both WGMs are TE-polarized. The solid curves correspond to the electric field at the microsphere’s surface. (**b**) Spatial distributions of the total potential ${U}_{\mathrm{t}\mathrm{o}\mathrm{t}}\left(\rho >R,\theta ,\varphi \right)$. The three-dimensional trapping with a potential depth of 32 µK is achieved by setting the powers of red- and blue-detuned WGM fields at ${I}_{\mathrm{r}}=27$ mW and ${I}_{\mathrm{b}}=87$ mW, respectively. The optical trap presents a ring pattern with 2*m*_r_ lattice sites in the $x-y$ plane.

In^26^, it shows that the red-detuned magic *λ*_r_, at which ${\alpha }_{\mathrm{S}}\left({\lambda }_{\mathrm{r}},{\theta }_{\text{B}}\right)={\alpha }_{\mathrm{P}}\left({\lambda }_{\mathrm{r}},{\theta }_{\text{B}}\right)$, may be situated in the range from 810 to 930 nm, depending on *θ*_B_. We choose the standing-wave WGM as $\left({n}_{\mathrm{r}}=1,{l}_{\mathrm{r}}=49,\pm {m}_{\mathrm{r}}\right)$ with ${m}_{\mathrm{r}}=47$ and obtain ${\lambda }_{\mathrm{r}}=832.69$ nm from Eq. (9). Figure 2a illustrates the corresponding light-field distribution, where $\left({l}_{\mathrm{r}}-{m}_{\mathrm{r}}+1\right)$ maxima are presented in the plane perpendicular to the equator of the microsphere while the light field exhibits a ring-shaped lattice pattern in the equatorial plane with the number of lattice sites equal to 2*m*_r_. It is found that the polarizability difference $\mathrm{\Delta }{\alpha }_{\mathrm{r}}\left({\lambda }_{\mathrm{r}},{\theta }_{\text{B}}\right)={\alpha }_{\mathrm{S}}\left({\lambda }_{\mathrm{r}},{\theta }_{\text{B}}\right)-{\alpha }_{\mathrm{P}}\left({\lambda }_{\mathrm{r}},{\theta }_{\text{B}}\right)$ is cancelled at ${\theta }_{\text{B}}={55.45}^{^\circ }$ (see Methods). The corresponding atomic polarizability is evaluated as ${\alpha }_{\mathrm{r}}\equiv {\alpha }_{\mathrm{S}}\left({\lambda }_{\mathrm{r}},{\theta }_{\text{B}}\right)={\alpha }_{\mathrm{P}}\left({\lambda }_{\mathrm{r}},{\theta }_{\text{B}}\right)=0.27$ a.u. (atomic units) and one has the red-detuned optical potential.

12 $${U}_{\mathrm{r}}\left(\rho >R,\theta ,\varphi \right)\equiv {U}_{\mathrm{r}}^{\left(\mathrm{S}\right)}\left(\rho >R,\theta ,\varphi \right)={U}_{\mathrm{r}}^{\left(\mathrm{P}\right)}\left(\rho >R,\theta ,\varphi \right).$$

That is, the atom experiences the same red-detuned optical potential when it is in $\left| S \right\rangle$ and $\left| P \right\rangle$. The rates of change of $\mathrm{\Delta }{\alpha }_{\mathrm{r}}\left({\lambda }_{\mathrm{r}},{\theta }_{\text{B}}\right)$ with respect to *λ*_r_ and *θ*_B_ are computed as $\partial \mathrm{\Delta }{\alpha }_{\mathrm{r}}\left({\lambda }_{\mathrm{r}},{\theta }_{\text{B}}\right)/\partial {\lambda }_{\mathrm{r}}=-7.76\times {10}^{-4}$ a.u. per nm and $\partial \mathrm{\Delta }{\alpha }_{\mathrm{r}}\left({\lambda }_{\mathrm{r}},{\theta }_{\text{B}}\right)/\partial {\theta }_{\text{B}}=1.73\times {10}^{-4}$ a.u. per millidegree, respectively. The current optical and atomic physics techniques ensure that the influence of the intensity fluctuations of the trapping beams and the misalignment of the magnetic field direction on the intercombination transition of ^88^Sr can be well below its intrinsic linewidth *γ*.

In addition, as demonstrated in^39^, the blue-detuned magic wavelength may be found around 390 nm. Thus, we choose the travelling WGM as $\left({n}_{\mathrm{b}}=1,{l}_{\mathrm{b}}=109,{m}_{\mathrm{b}}=109\right)$ with ${\lambda }_{\mathrm{b}}=392.58$ nm. The corresponding light-field distribution has only one maximum in the polar direction and is independent of the azimuth angle (Fig. 2a). At *λ*_b_ and ${\theta }_{\text{B}}=55.45^\circ$, one obtains the dynamic polarizabilities ${\alpha }_{\mathrm{S}}\left({\lambda }_{\mathrm{b}},{\theta }_{\text{B}}\right)=-0.48$ a.u. and ${\alpha }_{\mathrm{P}}\left({\lambda }_{\mathrm{b}},{\theta }_{\text{B}}\right)=-0.49$ a.u. when the atom is in $\left| S \right\rangle$ and $\left| P \right\rangle$, respectively. The polarizability difference $\mathrm{\Delta }{\alpha }_{\mathrm{b}}\left({\lambda }_{\mathrm{b}},{\theta }_{\text{B}}\right)={\alpha }_{\mathrm{S}}\left({\lambda }_{\mathrm{b}},{\theta }_{\text{B}}\right)-{\alpha }_{\mathrm{P}}\left({\lambda }_{\mathrm{b}},{\theta }_{\text{B}}\right)$ is about 0.01 a.u. with the rates of change $\partial \mathrm{\Delta }{\alpha }_{\mathrm{b}}\left({\lambda }_{\mathrm{b}},{\theta }_{\text{B}}\right)/\partial {\lambda }_{\mathrm{b}}=1.2\times {10}^{-3}$ a.u. per nm and $\partial \mathrm{\Delta }{\alpha }_{\mathrm{b}}\left({\lambda }_{\mathrm{b}},{\theta }_{\text{B}}\right)/\partial {\theta }_{\text{B}}=1.5\times {10}^{-5}$ a.u. per millidegree. Due to the small relative polarizability difference $\mathrm{\Delta }{\alpha }_{\mathrm{b}}\left({\lambda }_{\mathrm{b}},{\theta }_{\text{B}}\right)/{\alpha }_{\mathrm{S},\mathrm{P}}\left({\lambda }_{\mathrm{b}},{\theta }_{\text{B}}\right)\sim -2.1\times {10}^{-2}$, one may approximate ${\alpha }_{\mathrm{b}}\equiv {\alpha }_{\mathrm{S}}\left({\lambda }_{\mathrm{b}},{\theta }_{\text{B}}\right)\approx {\alpha }_{\mathrm{P}}\left({\lambda }_{\mathrm{b}},{\theta }_{\text{B}}\right)$ and obtain.

13 $${U}_{\mathrm{b}}\left(\rho >R,\theta ,\varphi \right)\equiv {U}_{\mathrm{b}}^{\left(\mathrm{S}\right)}\left(\rho >R,\theta ,\varphi \right)\approx {U}_{\mathrm{b}}^{\left(\mathrm{P}\right)}\left(\rho >R,\theta ,\varphi \right),$$

i.e., the atom experiences approximately the same blue-detuned optical potential when it is in $\left| S \right\rangle$ and $\left| P \right\rangle$.

Besides optical potentials ${U}_{\mathrm{b},\mathrm{r}}\left(\rho >R,\theta ,\varphi \right)$, van der Walls potentials.

14 $${U}_{\text{vdW}}^{\left(j=\mathrm{S},\mathrm{P}\right)}\left(\rho >R\right)=-{C}_{3}^{\left(j\right)}/{\left(\rho -R\right)}^{3},$$

are also exerted on atoms near the microsphere’s surface. The coefficients ${C}_{3}^{\left(j=\mathrm{S},\mathrm{P}\right)}$ can be computed by^27^ ${C}_{3}^{\left(j\right)}=\frac{\hslash }{16{\mathrm{\pi }}^{2}{\varepsilon }_{0}}{\int }_{0}^{\infty }{\alpha }_{j}\left(i\xi \right)\frac{\epsilon \left(i\xi \right)-1}{\epsilon \left(i\xi \right)+1}d\xi$ and we have ${C}_{3}^{\left(\mathrm{S}\right)}=722.87$ Hz µm^−3^ and ${C}_{3}^{\left(\mathrm{P}\right)}=744.24$ Hz µm^−3^. Due to the small relative difference $({C}_{3}^{\left(\mathrm{S}\right)}-{C}_{3}^{\left(\mathrm{P}\right)})/{C}_{3}^{\left(\mathrm{S},\mathrm{P}\right)}\sim -3.0\times {10}^{-2}$, one may approximate.

15 $${U}_{\text{vdW}}\left(\rho >R\right)\equiv {U}_{\text{vdW}}^{\left(\mathrm{S}\right)}\left(\rho >R\right)\approx {U}_{\text{vdW}}^{\left(\mathrm{P}\right)}\left(\rho >R\right).$$

Consequently, the total potential exerted on the atom in $\left| S \right\rangle$ and $\left| P \right\rangle$ is expressed as:

16 $${U}_{\mathrm{t}\mathrm{o}\mathrm{t}}\left(\rho >R,\theta ,\varphi \right)={U}_{\mathrm{b}}\left(\rho >R,\theta ,\varphi \right)+{U}_{\mathrm{r}}\left(\rho >R,\theta ,\varphi \right)+{U}_{\text{vdW}}\left(\rho >R\right).$$

The trapping beam powers are given by ${I}_{u=\mathrm{b},\mathrm{r}}=\left(c{\varepsilon }_{0}/2\right)\iint {\stackrel{\sim }{\epsilon }}_{u}\left(\rho \right){\left|{\mathbf{E}}_{u}\left(\rho ,\theta ,\varphi =0\right)\right|}^{2}\rho d\rho \mathrm{sin}\theta d\theta$ with the relative permittivity ${\stackrel{\sim }{\epsilon }}_{u}\left(\rho <R\right)=\epsilon \left({\lambda }_{u}\right)$ and ${\stackrel{\sim }{\epsilon }}_{u}\left(\rho >R\right)=1$. Adjusting ${I}_{\mathrm{b},\mathrm{r}}$ allows a three-dimensional trapping of ^88^Sr atoms in the vicinity of the microsphere’s surface.

Figure 2b displays the distribution of the lattice potential with ${I}_{\mathrm{r}}=27$ mW and ${I}_{\mathrm{b}}=87$ mW. There exists a potential well with a depth of 32 µK, large enough for trapping ultracold ^88^Sr atoms (temperature 0.4 µK^40^), in the plane perpendicular to the equator of the microsphere. The distance from the microsphere’s surface to the potential-well minimum is $\left({\rho }_{0}-R\right)=137$ nm with the distance ${\rho }_{0}$ between the microsphere’s center and the potential well. In the equatorial plane, the trapping potential presents a ring-shaped lattice pattern and the number of lattice sites is 2*m*_r_. Solving the Schrödinger equation of an atom moving in an optical lattice site, one may derive the vibration frequencies ${\mathrm{\Omega }}_{\rho }=2\mathrm{\pi }\times 86$ kHz, ${\mathrm{\Omega }}_{\theta }=2\mathrm{\pi }\times 25$ kHz and ${\mathrm{\Omega }}_{\varphi }=2\mathrm{\pi }\times 145$ kHz along ${\text{e}}_{\rho }$, ${\text{e}}_{\theta }$ and ${\text{e}}_{\varphi }$, respectively (see Methods). The corresponding Lamb–Dicke parameters are $\left\{{\eta }_{i=\rho ,\theta ,\varphi }={k}_{0}\sqrt{\hslash /2M{\mathrm{\Omega }}_{i}}\right\}=\left\{\mathrm{0.23,0.43,0.18}\right\}$ with the mass of atom *M*. In the Lamb–Dicke regime, ${\eta }_{i=\rho ,\theta ,\varphi }^{2}\ll 1$, the transition of an atom between two internal states $\left| S \right\rangle$ and $\left| P \right\rangle$ hardly affects its external (motional/vibrational) states, i.e., free of photon-recoil shifts. The lifetime of a confined atom in $\left| S \right\rangle$ is determined by the rate of the atom scattering the trapping-beam photons and reaches as long as 10^3^ s (see Methods). By contrast, the lifetime of a confined atom in $\left| P \right\rangle$ is primarily limited by the intrinsic decay rate *γ* of the intercombination transition. Finally, it is worth noting that the ring-shaped lattice pattern shown in Fig. 2b is similar to the stationary interference pattern of the composite WGMs that have different wavelengths but share the same resonance frequency^41–43^.

### Strong atom-microcavity coupling

We now consider the atom-microcavity interaction. The coupling strength between the lasing $\left({n}_{0},{l}_{0},\pm {m}_{0}\right)$ WGMs and the $\left| S \right\rangle$–$\left| P \right\rangle$ transition of ^88^Sr is given by.

17 $$g=\sqrt{F\kappa \gamma }/2,$$

where *F* is the so-called Purcell factor,

18 $$F=\frac{3Q}{4{\mathrm{\pi }}^{2}}\left({\lambda }_{0}^{3}/{V}_{\text{eff}}\right){\mathrm{cos}}^{2}{\theta }_{\mathrm{B}},$$

$Q={\omega }_{0}/\kappa$ denotes the quality factor of the lasing WGMs with the total photon loss rate *κ*, and ${V}_{\text{eff}}$ gives the effective mode volume,

19 $${V}_{\text{eff}}=\int {\stackrel{\sim }{\epsilon }}_{0}\left(\rho \right){\left|{\mathbf{E}}_{0}\left(\mathbf{r}\right)\right|}^{2}d\mathbf{r}/{\left|{\mathbf{E}}_{0}\left({\mathbf{r}}_{\mathrm{a}}\right)\right|}^{2},$$

with the WGM light field ${\mathbf{E}}_{0}\left(\mathbf{r}\right)$ and the location ${\mathbf{r}}_{\mathrm{a}}$ of an atom in a lattice site. For the lattice potential shown in Fig. 2b, *V*_eff_ is evaluated as 827 µm^3^, which is ten thousand times smaller than that of a common Fabry–Pérot cavity^44^ and results in $g=2\pi \times 2.8$ MHz. The corresponding saturation photon number is extremely small, ${N}_{\mathrm{p}}^{\mathrm{s}}=2\times {10}^{-6}$.

The strong atom-cavity coupling requires $Q$ to be high enough that ${N}_{\mathrm{a}}^{\mathrm{c}}\ll 1$. The quality factor of the lasing $\left({n}_{0},{l}_{0},\pm {m}_{0}\right)$ WGMs is given by.

20 $${Q}^{-1}={Q}_{\mathrm{r}\mathrm{a}\mathrm{d}}^{-1}+{Q}_{\mathrm{m}\mathrm{a}\mathrm{t}}^{-1}+{Q}_{\mathrm{s}\mathrm{s}}^{-1}+{Q}_{\mathrm{s}\mathrm{a}}^{-1}+{Q}_{\mathrm{c}}^{-1},$$

where ${Q}_{\mathrm{r}\mathrm{a}\mathrm{d}}$, ${Q}_{\mathrm{m}\mathrm{a}\mathrm{t}}$, ${Q}_{\mathrm{s}\mathrm{s}}$, ${Q}_{\mathrm{s}\mathrm{a}}$, and ${Q}_{\mathrm{c}}$ quantify the optical loss mechanisms of radiation, material attenuation, surface scattering, surface adsorption, and microsphere-fiber coupling, respectively. The radiation loss is attributed to the fact that a portion of intracavity light leaks out of the microsphere each time when the light beam hits on the microsphere’s surface. The relevant quality factor is evaluated by^45^.

21 $${Q}_{\mathrm{r}\mathrm{a}\mathrm{d}}=\frac{2{l}_{0}+1}{4}\sqrt{\frac{\epsilon \left({\lambda }_{0}\right)-1}{\epsilon \left({\lambda }_{0}\right)}}\mathrm{exp}\left[\left(2{l}_{0}+1\right)\left({\beta }_{{n}_{0},{l}_{0}}-\mathrm{tanh}{\beta }_{{n}_{0},{l}_{0}}\right)\right],$$

with the shorthand

22 $${\beta }_{{n}_{0},{l}_{0}}={\mathrm{cosh}}^{-1}\left\{\sqrt{\epsilon \left({\lambda }_{0}\right)}{\left[1+\frac{2}{2{l}_{0}+1}\left({u}_{{n}_{0}}{\left(\frac{2{l}_{0}+1}{4}\right)}^{1/3}-\sqrt{\frac{\epsilon \left({\lambda }_{0}\right)}{\epsilon \left({\lambda }_{0}\right)-1}}\right)\right]}^{-1}\right\},$$

and the ${n}_{0}$-th root ${u}_{{n}_{0}}$ of the Airy function $\mathrm{Ai}\left(-u\right)$. The radiation-loss-limited quality factor is computed as ${Q}_{\mathrm{r}\mathrm{a}\mathrm{d}}=1.5\times {10}^{8}$, much lower than that of a typical microsphere^16^. This is because the microsphere’s radius *R* here is substantially reduced so as to enhance the evanescent field and suppress the effective mode volume *V*_eff_. The material-loss-limited ${Q}_{\mathrm{m}\mathrm{a}\mathrm{t}}$ takes the form.

23 $${Q}_{\mathrm{m}\mathrm{a}\mathrm{t}}=\left(2\pi/{\lambda }_{0}\right)\sqrt{\epsilon \left({\lambda }_{0}\right)}/{\alpha }_{\mathrm{m}\mathrm{a}\mathrm{t}},$$

with the attenuation coefficient ${\alpha }_{\mathrm{m}\mathrm{a}\mathrm{t}}$. Generally, ${\alpha }_{\mathrm{m}\mathrm{a}\mathrm{t}}$ of the silica is composed of the Rayleigh scattering and the material absorption. At ${\lambda }_{0}$, ${\alpha }_{\mathrm{m}\mathrm{a}\mathrm{t}}$ approximates^46^ 6 dB km^−1^ and ${Q}_{\mathrm{m}\mathrm{a}\mathrm{t}}$ is estimated as $1.1\times {10}^{10}$. The surface-roughness-limited ${Q}_{\mathrm{s}\mathrm{s}}$ may be evaluated by.

24 $${Q}_{\mathrm{s}\mathrm{s}}=\frac{3\sqrt{\epsilon \left({\lambda }_{0}\right)}}{4{\mathrm{\pi }}^{2}}{\left(\frac{\epsilon \left({\lambda }_{0}\right)}{\epsilon \left({\lambda }_{0}\right)-1}\right)}^{2}\frac{{\lambda }_{0}^{3}\sqrt{2R{\lambda }_{0}}}{{\sigma }^{2}{B}^{2}},$$

with the standard deviation *σ* and spatial correlation length *B* of the surface roughness of microsphere^17^. In experiment, *σ* and *B* are measured to be 2 nm and 5 nm, respectively. One has ${Q}_{\mathrm{s}\mathrm{s}}=3.5\times {10}^{8}$, which is also restricted by the small radius *R* of the microsphere. The optical loss that is caused by the adsorbed water upon the microsphere’s surface leads to the quality factor component.

25 $${Q}_{sa}=\sqrt{\frac{\mathrm{\pi }R}{16{n}_{wat}^{3}{\lambda }_{0}}}\frac{1}{\delta \beta },$$

with the thickness *δ* and absorption coefficient *β* of the water layer^17^. The refractive index of water *n*_wat_ has been listed in^47^. The typical value *δ* ~ 0.2 nm leads to ${Q}_{\mathrm{s}\mathrm{a}}=8\times {10}^{9}$.

The intracavity photons are extracted from the microcavity by using the same fiber-microcavity coupler. The external-coupling-limited ${Q}_{\mathrm{c}}$ can be adjusted by changing the distance between the microsphere and the tapered fiber. In the weak microsphere-fiber coupling limit, the total quality factor $Q$ is mainly limited by the radiation loss ${Q}_{\mathrm{r}\mathrm{a}\mathrm{d}}$ and the microcavity output power is suppressed. We assume $Q={10}^{8}$ with $\kappa =2\mathrm{\pi }\times 4.4$ MHz and the corresponding Purcell factor *F* reaches $\sim {10}^{3}$. The critical atom number is evaluated as ${N}_{\mathrm{a}}^{\mathrm{c}}={10}^{-3}$, much smaller than unity. By contrast, the relatively strong microsphere-fiber coupling with, for example, ${Q}_{\mathrm{c}}={10}^{7}$ limits the total $Q$ factor, which reduces *F* to $\sim {10}^{2}$ while increases ${N}_{\mathrm{a}}^{\mathrm{c}}$ to ${10}^{-2}$ (i.e., still well below unity) but enhances the microcavity output power. Therefore, the combined system operates in the strong coupling regime.

The dissipative dynamics of the atom-microsphere coupled system is governed by the master Eq.^48^.

26 $$\dot{\rho }=-i\left[H/\mathrm{\hslash },\rho \right]+{\mathcal{D}}_{\mathrm{c}\mathrm{a}\mathrm{v}}\left(\rho \right)+{\mathcal{D}}_{\mathrm{s}\mathrm{p}}\left(\rho \right),$$

where *ρ* is the combined density matrix and the Hamiltonian *H* describes the coherent interaction between atoms and two degenerate lasing WGMs.

27 $$H/\mathrm{\hslash }=g{\sum }_{u=1}^{N}\left[{\sigma }_{u}^{\dagger}\left({a}_{\mathrm{C}\mathrm{W}}{e}^{i{k}_{0}{\rho }_{0}{\varphi }_{u}}+{a}_{\mathrm{C}\mathrm{C}\mathrm{W}}{e}^{-i{k}_{0}{\rho }_{0}{\varphi }_{u}}\right)+h.c.\right],$$

with the creation ${a}_{i}^{\dagger}$ and annihilation ${a}_{i}$ operators for the $i=\mathrm{C}\mathrm{W},\mathrm{C}\mathrm{C}\mathrm{W}$ WGM and the raising ${\sigma }_{u}^{\dagger}={\left(\left| P \right\rangle\left\langle\mathrm{S}\right|\right)}_{u}$ and lowering ${\sigma }_{u}={\left(\left|\mathrm{S}\right\rangle\left\langle\mathrm{P}\right|\right)}_{u}$ for the $u$ th atom. Here, ${\varphi }_{u}$ denotes the azimuth angle at the position of the $u$ th atom and *N* gives the number of trapped atoms. The dissipation terms ${\mathcal{D}}_{\mathrm{c}\mathrm{a}\mathrm{v}}\left(\rho \right)$ and ${\mathcal{D}}_{\mathrm{s}\mathrm{p}}\left(\rho \right)$ account for the photon loss and the spontaneous decay of $\left| P \right\rangle$-populated atoms, respectively, and take the Lindblad form.

28 $${\mathcal{D}}_{\mathrm{c}\mathrm{a}\mathrm{v}}\left(\rho \right)=\left(\kappa /2\right){\sum }_{i=\mathrm{C}\mathrm{W},\mathrm{C}\mathrm{C}\mathrm{W}}\left(2{a}_{i}\rho {a}_{i}^{\dagger}-{a}_{i}^{\dagger}{a}_{i}\rho -\rho {a}_{i}^{\dagger}{a}_{i}\right),$$

29 $${\mathcal{D}}_{\mathrm{s}\mathrm{p}}\left(\rho \right)=\left(\gamma /2\right){\sum }_{u=1}^{N}\left(2{\sigma }_{u}\rho {\sigma }_{u}^{\dagger}-{\sigma }_{u}^{\dagger}{\sigma }_{u}\rho -\rho {\sigma }_{u}^{\dagger}{\sigma }_{u}\right).$$

To solve the master equation, we choose the Hilbert space to be spanned by an orthonormal set of product states, $\left\{ {\left| \psi  \right._{1}  \otimes  \ldots  \otimes \left| \psi  \right._{N}  \otimes \left| {n_{{{\text{CW}}}} } \right. \otimes \left| {n_{{{\text{CCW}}}} } \right.;\psi  = {\text{S}},{\text{P}};n_{{i = {\text{CW}},{\text{CCW}}}}  = 0,1,2, \ldots } \right\}$. The master equation can be numerically simulated by using the Monte Carlo wave-function (MCWF) method^49^. Due to the limited computer memory, we restrict ourselves to the system with up to *N* = 7 atoms, i.e., less than ten percent lattice sites are occupied.

### Spontaneous emission in microcavity

As pointed out in^50^, the spontaneous emission of an atom depends strongly on the environment it resides in. The environment may be tailored by an optical cavity and the resultant atomic fluorescence is directional and spectrally broadened by the Purcell factor *F*. Let us assume that all trapped atoms are initially prepared in $\left| P \right\rangle$ and none intracavity photons exist. In addition, each lattice site is occupied by at most one atom. Using the MCWF approach, we simulate the time evolutions of intracavity photon numbers ${N}_{\mathrm{p},i=\mathrm{C}\mathrm{W},\mathrm{C}\mathrm{C}\mathrm{W}}\left(t\right)=\mathrm{Tr}\left[\rho \left(t\right){a}_{i}^{\dagger}{a}_{i}\right]$, the intermode correlation ${C}_{\mathrm{p}}\left(t\right)=\left|\mathrm{Tr}\left[\rho \left(t\right){a}_{\mathrm{C}\mathrm{W}}^{\dagger}{a}_{\mathrm{C}\mathrm{C}\mathrm{W}}\right]\right|$, the number of $\left|P\right\rangle$-populated atoms ${N}_{\mathrm{a}}\left(t\right)={\sum }_{u=1}^{N}\mathrm{Tr}\left[\rho \left(t\right){\sigma }_{u}^{\dagger}{\sigma }_{u}\right]$, and the interatomic correlation ${C}_{\mathrm{a}}\left(t\right)=\frac{1}{N\left(N-1\right)}\left|{\sum }_{\mu \ne \nu }\mathrm{Tr}\left[\rho \left(t\right){\sigma }_{u}^{\dagger}{\sigma }_{\nu }\right]\right|$.

**Figure 3:**
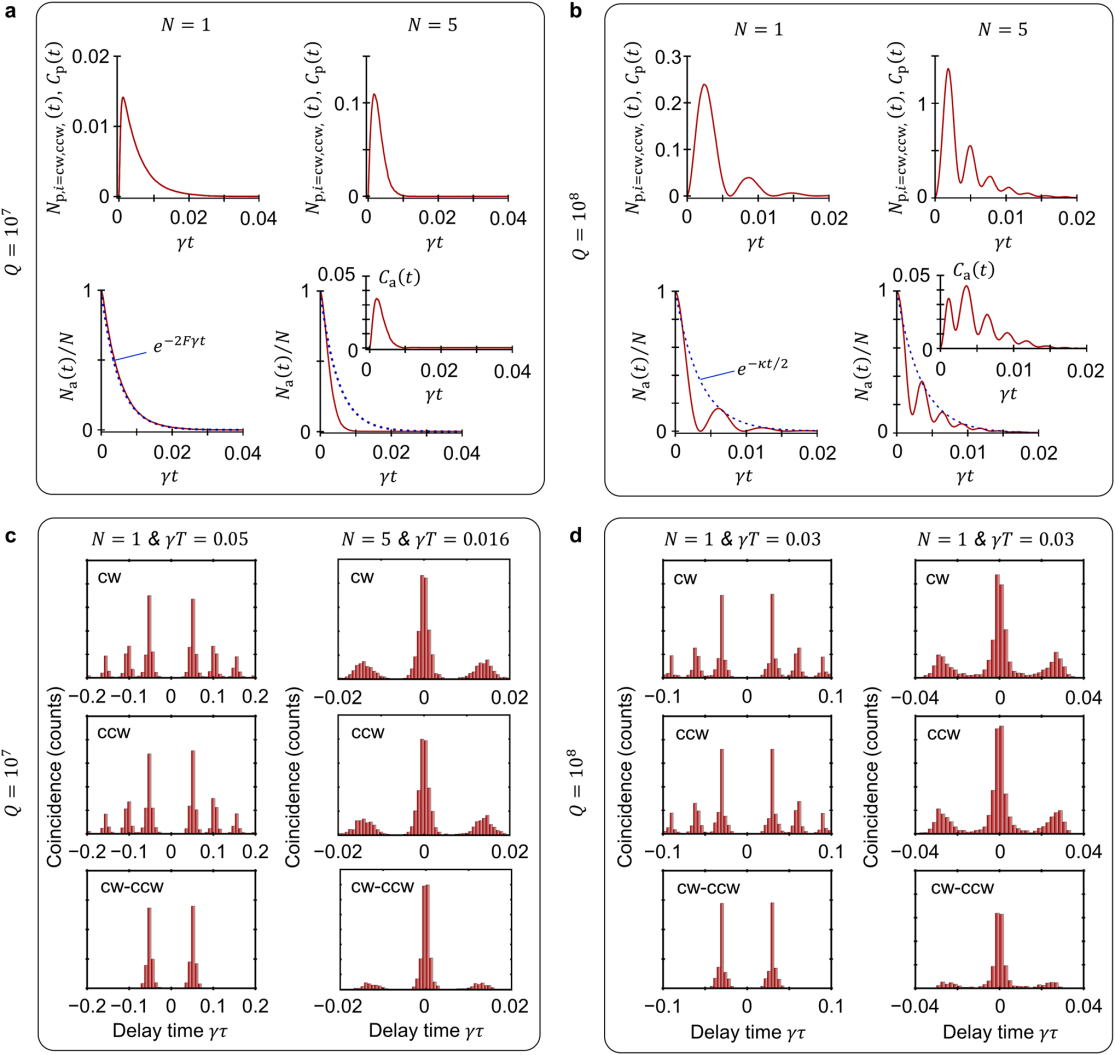
Spontaneous emission of *N* atoms confined in the vicinity of a microsphere. (**a**) Intracavity photon numbers ${N}_{\mathrm{p},i=\mathrm{C}\mathrm{W},\mathrm{C}\mathrm{C}\mathrm{W}}\left(t\right)$, intermode correlation ${C}_{\mathrm{p}}\left(t\right)$, atomic population ${N}_{\mathrm{a}}\left(t\right)$ in $\left| P \right\rangle$ and interatomic correlation ${C}_{\mathrm{a}}\left(t\right)$ of the coupled system with a fast ($Q={10}^{7}$, $\kappa =2\mathrm{\pi }\times 44$ MHz) microcavity. The corresponding Purcell factor is $F={10}^{2}$. All atoms are initially prepared in $\left| P \right\rangle$ and the microcavity contains zero photons. (**b**) Time evolutions of ${N}_{\mathrm{p},i=\mathrm{C}\mathrm{W},\mathrm{C}\mathrm{C}\mathrm{W}}\left(t\right)$, ${C}_{\mathrm{p}}\left(t\right)$, ${C}_{\mathrm{a}}\left(t\right)$ and ${N}_{\mathrm{a}}\left(t\right)$ for the system in the slow-microcavity regime with $Q={10}^{8}$. (**c**,**d**) Coincidence statistics of the Hanbury Brown–Twiss detection, where the atoms are repeatedly prepared in $\left| P \right\rangle$ by a sequence of short resonant light π-pulses. The period *T* of light pulse sequence is set at $\gamma T=0.05$ for *N* = 1 and $\gamma T=0.016$ for *N* = 5 ($\gamma T=0.03$ for both *N* = 1 and *N* = 5) in the fast-microcavity (slow-microcavity) regime. All plots are obtained by using the MCWF method.

The results have been presented in Fig. 3. It is seen that ${N}_{\mathrm{p},\mathrm{C}\mathrm{W}}\left(t\right)={N}_{\mathrm{p},\mathrm{C}\mathrm{C}\mathrm{W}}\left(t\right)={C}_{\mathrm{p}}\left(t\right)$, i.e., the atoms contribute equally to two degenerate lasing WGMs and the intermode correlation is as strong as the field intensity of individual modes. In the fast-cavity regime (e.g., $Q={10}^{7}$ with $\kappa \gg g\gg \gamma$), the photons emitted by atoms rapidly escape from the microcavity well before being reabsorbed by atoms. Consequently, ${N}_{\mathrm{a}}\left(t\right)$ degrades monotonically with a decay constant (defined by the reciprocal of a time length after ${N}_{\mathrm{a}}\left(t\right)$ falls to 1/*e* of its initial value) of about 2*Fγ*. The extra factor 2 comes from the fact that atoms interact with two lasing WGMs. For the one-atom system (see Fig. 3a with *N* = 1), the decay behavior of ${N}_{\mathrm{a}}\left(t\right)$ well matches the ideal exponential decay $\mathrm{exp}\left(-2F\gamma t\right)$, i.e., Lorentzian broadening. As the atom number *N* is increased, the decay of ${N}_{\mathrm{a}}\left(t\right)$ is apparently accelerated (see Fig. 3a with *N* = 5). This is because the exchange of intracavity photons among different atoms gives rise to the nonzero interatomic correlation ${C}_{\mathrm{a}}\left(t\right)$, leading to the partially constructive interference of multiple emitters, i.e., superradiance. For a slow microcavity (e.g., $Q={10}^{8}$ with $\kappa \sim g\gg \gamma$), both ${N}_{\mathrm{p},i=\mathrm{C}\mathrm{W},\mathrm{C}\mathrm{C}\mathrm{W}}\left(t\right)$ and ${N}_{\mathrm{a}}\left(t\right)$ exhibit an oscillatory behavior (Fig. 3b), i.e., the Purcell picture breaks down, since the atoms can reabsorb intracavity photons before the photons escape from the microcavity. The envelope of ${N}_{\mathrm{a}}\left(t\right)$ follows the exponential decay with a decay constant given by the half of the microcavity loss rate $\kappa /2$. Moreover, we exam the efficiency of the microcavity collecting the fluorescence of atoms $\eta ={\sum }_{i=\mathrm{C}\mathrm{W},\mathrm{C}\mathrm{C}\mathrm{W}}\int {N}_{\mathrm{p},i}\left(t\right)dt/N$ and find that for both fast and slow microcavities, *η* reaches almost unity because of $g\gg \gamma$. Such a high collection efficiency is of particularly useful for the applications in optical quantum information processing^51^.

We further assume that the atoms are repeatedly prepared in $\left| P \right\rangle$ with a period *T* and perform the Hanbury Brown–Twiss measurement on the photon emission, i.e., the coincidence counts as a function of the time delay *τ* between two successive photon detection events^52^. The duration *T* is long enough (i.e., *FγT* ≫ 1 for the fast microcavity while *κT* ≫ 1 for the slow microcavity) that the atoms decay sufficiently at the end of each period. For the one-atom system with *N* = 1, the self-coincidence statistics of individual WGM outputs is almost zero at $\tau =0$ (Fig. 3c,d). In other words, the system cannot emit more than one CW or CCW photon simultaneously. Such a purely nonclassical behavior is expectable since the system is initialized with only one energy quantum within each period *T*. Also, the cross-coincidence statistics between two WGMs reaches zero at the zero-time delay $\tau =0$. That is, the system cannot emit a pair of CW and CCW photons at the same time, which, however, is not a nonclassical property^53^. The one-atom operation may be of particular use for an efficient single-photon emitter. For the system with *N* > 1, either self-coincidence or cross-coincidence statistics presents a peak at the zero-time delay $\tau =0$, denoting that two lasing WGMs emit bunched photons.

### Microlaser

The atom-microcavity coupled system can operate as a three-level microlaser. The pump process is implemented by using a pair of laser beams to resonantly drive the two-photon $\left| S \right\rangle$–(5*s*5*p*)^3^*P*_1_ (*m* =  − 1)–(5*s*6*s*)^3^*S*_1_ transition (Fig. 1b). Several repump beams are applied to excite the atoms in (5*s*5*p*)^3^*P*_0_, (5*s*5*p*)^3^*P*_1_ (*m* = 1) and (5*s*5*p*)^3^*P*_2_ to (5*s*6*s*)^3^*S*_1_. The atoms accumulate in $\left| P \right\rangle$ via the rapid spontaneous decay from (5*s*6*s*)^3^*S*_1_ to $\left| P \right\rangle$. To mathematically describe the pump process, an extra term^44^.

30 $${\mathcal{D}}_{\mathrm{p}\mathrm{u}\mathrm{m}\mathrm{p}}\left(\rho \right)=\left(\mathrm{\Gamma }/2\right){\sum }_{u=1}^{N}\left(2{\sigma }_{u}^{\dagger}\rho {\sigma }_{u}-{\sigma }_{u}{\sigma }_{u}^{\dagger}\rho -\rho {\sigma }_{u}{\sigma }_{u}^{\dagger}\right),$$

with an effective pumping rate Γ should be added into the master Eq. (26). Actually, ${\mathcal{D}}_{\mathrm{p}\mathrm{u}\mathrm{m}\mathrm{p}}\left(\rho \right)$ is identical to ${\mathcal{D}}_{\mathrm{s}\mathrm{p}}\left(\rho \right)$ with the replacements $\gamma \to \mathrm{\Gamma }$, ${\sigma }_{u}\to {\sigma }_{u}^{\dagger}$, and ${\sigma }_{u}^{\dagger}\to {\sigma }_{u}$. Again, the MCWF method is applied to solve the lasing dynamics.

**Figure 4:**
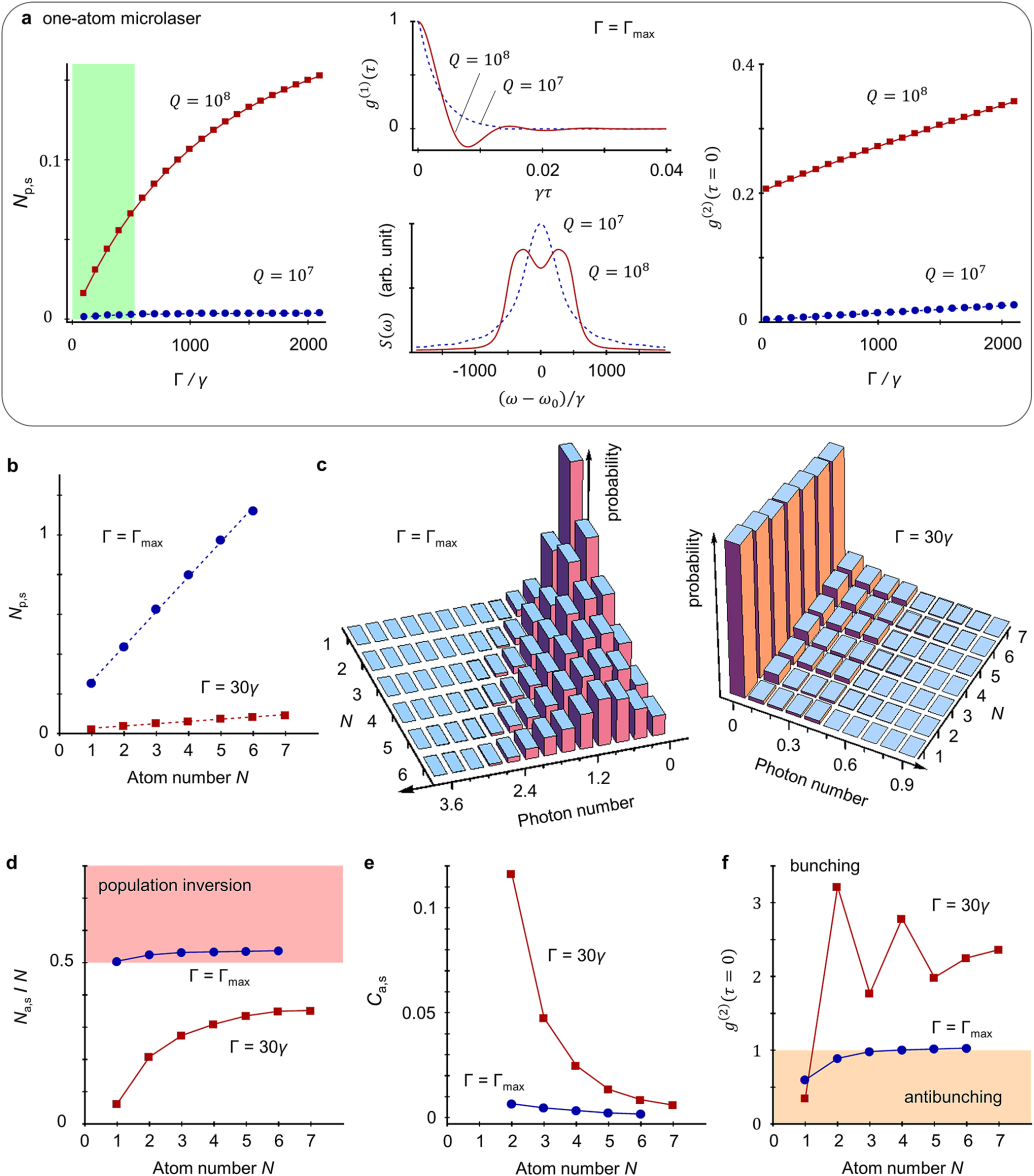
Microlasing action. (**a**) Steady-state intracavity photon number *N*_p,s_ as a function of the pump rate Γ, first-order correlation ${g}^{\left(1\right)}\left(\tau \right)$ and laser spectrum $S\left(\omega \right)$ at the maximum pump Γ = Γ_max_, and zero-time-delay intensity correlation ${g}^{\left(2\right)}\left(\tau =0\right)$ of one-atom microlaser with *N* = 1. For the low-$Q$ microcavity with $Q={10}^{7}$, the linewidth (full width half maximum) of $S\left(\omega \right)$ is about $633\gamma$, much smaller than the intrinsic decay rate *κ* of WGMs. For the high-$Q$ microcavity with $Q={10}^{8}$, the spectral linewidth of either peak in $S\left(\omega \right)$ is evaluated by fitting the function $G\left(\tau \right)=A{e}^{-\mathrm{\Delta }\tau /2}\mathrm{cos}\left(\delta \tau +\theta \right)$ with the amplitude $A$, linewidth $\mathrm{\Delta }$, frequency shift $\delta$ and phase bias $\theta$ to the corresponding ${g}^{\left(1\right)}\left(\tau \right)$. The resultant linewidth is given by $\mathrm{\Delta }=475\gamma$, less than the decay rate *κ* of WGMs. (**b**) *N*_p,s_ as a function of the atom number *N* with Γ = Γ_max_ (filled circle symbol) and 30*γ* (filled square symbol). The dashed lines correspond to the linear curve-fitting results ${N}_{p,s}=kN+b$, where $\left(k,b\right)=\left(\mathrm{0.18,0.1}\right)$ for Γ = Γ_max_ and $\left(k,b\right)=\left(\mathrm{0.011,0.016}\right)$ for Γ = 30*γ*. (**c**) Distribution of intracavity photon number for different *N*. (**d**) Dependence of $\left| P \right\rangle$-population *N*_a,s_ on the atom number *N*. (**e**) Interatomic correlation *C*_a,s_ vs *N*. (**f**) Intensity correlation ${g}^{\left(2\right)}\left(\tau =0\right)$ changing with *N*. For (**b**–**f**), the quality factor of microsphere is $Q={10}^{8}$.

We focus on the steady-state (denoted by the subscript s) solutions, i.e., $\rho \left(t\to \infty \right)$. Due to the degeneracy of two lasing WGMs, these two WGMs have the same intracavity photon number, ${N}_{\mathrm{p},\mathrm{s}}=\mathrm{Tr}\left[{a}_{i=\mathrm{C}\mathrm{W},\mathrm{C}\mathrm{C}\mathrm{W}}^{\dagger}{a}_{i}\rho \left(t\to \infty \right)\right]$. For the one-atom microlaser with *N* = 1, *N*_p,s_ grows as Γ is increased (see Fig. 4a). However, Γ cannot be arbitrarily high since the spontaneous decay rate of ^88^Sr from (5*s*6*s*)^3^*S*_1_ to $\left| P \right\rangle$ sets the upper limit to Γ, i.e., ${\mathrm{\Gamma }}_{\text{max}}=2.5\times {10}^{7}$ s^−1^. Within the regime of Γ ≤ Γ_max_, *N*_p,s_ is well below unity. Enhancing *N*_p,s_ requires a larger $Q$ of microsphere, which is primarily restricted by the radiation loss (due to the small radius of microsphere). The laser fields are featured by their high temporal coherence that is characterized by the first-order correlation function, ${g}^{\left(1\right)}\left(\tau \right)=\langle{a}_{i=\mathrm{C}\mathrm{W},\mathrm{C}\mathrm{C}\mathrm{W}}^{\dagger}\left(t+\tau \right){a}_{i}\left(t\right)\rangle$ with a time delay *τ*. Here, *O*(*t*) denotes the operator *O* in the Heisenberg picture. The MCWF method is also applicable to simulate the two-time correlation function. It is seen from Fig. 4a that ${g}^{\left(1\right)}\left(\tau \right)$ monotonically degrades with *τ* for a low-$Q$ (e.g., ~ 10^7^) microcavity while ${g}^{\left(1\right)}\left(\tau \right)$ exhibits a damped oscillation behavior for a large $Q$ (e.g., ~ 10^8^).

We then map ${g}^{\left(1\right)}\left(\tau \right)$ onto the laser spectrum $S\left(\omega \right)$ via the Fourier transform, $S\left(\omega \right)=\int {g}^{\left(1\right)}\left(\tau \right){e}^{-i\omega \tau }d\tau$. For the low-$Q$ microcavity, $S\left(\omega \right)$ is single peaked (Fig. 4a) and the spectral linewidth (defined by the full width at half maximum) is much narrower than the WGM linewidth *κ*, indicating that highly coherent intracavity fields are established. By contrast, two spectral peaks are presented in $S\left(\omega \right)$ for the high-$Q$ microcavity (Fig. 4a), which is indeed ascribed to the vacuum Rabi splitting (i.e., the dressed-state picture) of the laser transition in the strong atom-microcavity coupling limit^54^. Remarkably, the linewidth of each spectral peak is close to the WGM linewidth *κ*, i.e., the temporal coherence of laser fields approaches that of cavity modes. We attribute this to the cavity pulling effect caused by the vacuum Rabi splitting. The high intensity (photon-number) fluctuations of intracavity fields lead to a strong spectral broadening. It should be noted that such a doublet vanishes for a high enough Γ because the dressed states with higher photon numbers are occupied^54^. Moreover, we perform the MCWF simulation on the second-order correlation function ${g}^{\left(2\right)}\left(\tau \right)=\langle{a}_{i=\mathrm{C}\mathrm{W},\mathrm{C}\mathrm{C}\mathrm{W}}^{\dagger}\left(t\right){a}_{i}^{\dagger}\left(t+\tau \right){a}_{i}\left(t+\tau \right){a}_{i}\left(t\right)\rangle$ and find that the outputs of the one-atom microlaser show the antibunching behavior, i.e., ${g}^{\left(2\right)}\left(0\right)<1$. A low-$Q$ microcavity highlights such an effect.

The pump and repump beams inevitably induce the extra light shift of the laser transition. This may reduce the overlap between the motional states of the atom in $\left| S \right\rangle$ and $\left| P \right\rangle$, thereby suppressing the coupling strength $g$. Achieving the maximum pump rate Γ_max_ demands a deep saturation of the $\left| S \right\rangle$–(5*s*5*p*)^3^*P*_1_ (*m* =  − 1) transition, which results in a light shift of the $\left| S \right\rangle$–$\left| P \right\rangle$ transition much exceeding its natural linewidth *γ*. Thus, the pump rate Γ is further restricted by the pump-beam-induced light shift. We set the strength of the magnetic field **B** to be 1 Gs, leading to a Zeeman shift of (5*s*5*p*)^3^*P*_1_ (*m* =  − 1) of $2\mathrm{\pi }\times 2.1$ MHz. It is estimated that Γ can be as high as 30*γ* without introducing a light shift larger than *γ* to the laser transition.

We further investigate the multiple-atom lasing action with a high-$Q$ microcavity and, for the sake of comparison, the pump rate Γ is set at Γ_max_ and 30*γ*. As illustrated in Fig. 4b, increasing the atom number *N* (up to *N* = 7) approximately linearly raises the photon number *N*_p,s_, i.e., ${N}_{\mathrm{p},\mathrm{s}}\approx kN$, and the factor of proportionality *k* depends on Γ. The histogram of the distribution of intracavity photon number (Fig. 4c) indicates that for Γ = Γ_max_ the maximum of the photon number moves away from zero and the width of the distribution expands as *N* goes up. By contrast, for Γ = 30*γ* the distribution depends less on *N*. We also numerically calculate the spectra of multiple-atom lasers and find that the double-peaked spectrum disappears when *N* ≥ 2. This is because multiple transitions between various dressed states contribute the intracavity fields. The linewidth of the laser spectrum does not show an apparent change as *N* is increased.

Interestingly, as plotted in Fig. 4d, the normalized $\left| P \right\rangle$-population of atoms ${N}_{\mathrm{a},\mathrm{s}}/N$ does not exceed one half for Γ = 30*γ*. That is to say, the population inversion is not a necessary condition for the lasing action, i.e., thresholdless. However, such a lasing without inversion is unlike that of^55,56^ since the latter originate from the quantum interference between multiple atomic transitions. Indeed, the laser dynamics here is an enhanced emission of photons under the strong atom-microcavity coupling condition. In addition, the steady-state interatomic correlation ${C}_{\mathrm{a},\mathrm{s}}$ strongly degrades as the system’s size grows (Fig. 4e). This behavior coincides with the mean-field conclusion derived in^44^^.^ One may expect ${C}_{\mathrm{a},\mathrm{s}}\approx 0$ for a macroscopic-sized system with $N\to \infty$, where the common mean-field theory is applicable. Surprisingly, Γ = 30*γ* gives a higher ${C}_{\mathrm{a},\mathrm{s}}$ than Γ = Γ_max_. That is, the less intracavity photon number enhances the interatomic correlation, although the latter arises from exchanging photons between atoms. For *N* = 1, the zero-time-delay ${g}^{\left(2\right)}\left(0\right)$ with Γ = 30*γ* is lower than that of Γ = Γ_max_ (Fig. 4f) because a photon emission event occurs well behind the previous event for a small Γ. When *N* > 1, the laser outputs the bunched photons and the photon-number fluctuations with Γ = 30*γ* are higher than that of Γ = Γ_max_.

## Discussion

We have studied an atom-microcavity platform, where a small number of neutral ^88^Sr atoms are confined in a ring-shaped lattice potential and strongly interact with a microsphere. The optical lattice is formed by combining the evanescent fields of two-color far-off-resonance WGMs. The red-detuned standing-wave WGM operates at a magic wavelength while the blue-detuned WGM wavelength is close to the other magic wavelength. Consequently, the lattice-induced light shift of the intercombination line of ^88^Sr is negligible compared to its natural linewidth *γ*. The resultant lattice potential can reach over 30 µK, deep enough for trapping ultracold atoms, and the trapping lifetime exceeds 10^3^ s, long enough for the continuous lasing action. The tiny mode volume and high quality factor of the microsphere ensure the system accessing the strong coupling regime. We exploit the function of the combined system as a microscale light source. The output vs. pump shows a thresholdless behavior, a direct result from the strong atom-microcavity coupling. The one-atom microlaser outputs the nonclassical light, potentially applicable in optical quantum information processing. The output fields become classical for the multi-atom operation.

Thus far, various laser schemes based on the forbidden ^1^*S*_0_–^3^*P*_0_ clock transition of alkaline-earth metal atoms have been proposed, such as optical lattice lasers^44^ and superradiant lasers^57^. Typically, the spectral linewidth of the gain medium is of the order of millihertz, extremely narrower than that of the cavity mode. According to the analysis in^58^, such bad-cavity lasers potentially work as active optical clocks that are immune to the influence of the thermal Brownian motion of mechanical cavities. The optical cavities used in these schemes are all in Fabry–Pérot structure, whose mode volume *V*_eff_ is artificially squeezed so as to strengthen the atom-cavity interaction. Nevertheless, *V*_eff_ is of the order of 10^−12^ m^3^. By contrast, WGM microcavities make use of the total internal reflection and possess ${V}_{\text{eff}}$ $\sim {10}^{-16}$ m^3^, leading to a two-orders-of-magnitude-enhanced coupling between atoms and microcavity. Although in this work we studied the lasing action based on the atomic intercombination transition (due to the limitation of computation time), the relevant analysis may be generalized to the (5*s*^2^)^1^*S*_0_–(5*s*5*p*)^3^*P*_0_ clock transition of neutral (fermionic) ^87^Sr. The hyperfine structure of ^87^Sr should be taken into account to find a WGM at, for example, the red-detuned magic wavelength^59^ under the condition that the other WGM is resonant to the clock transition. However, it is challenging to search for a third WGM, which operates at or close to a blue-detuned magic wavelength so that the induced light shift of the clock transition can be suppressed down to millihertz level.

Our platform with lattice-confined ultracold atoms also provides for various potential applications in many-body physics and quantum simulation and computation. The motion of atoms in a ring-shaped lattice matches strictly a one-dimensional many-body system with a periodic boundary condition that has been widely studied in literature. By contrast, the optical lattice formed by the standing wave of a retro-reflected laser beam actually has an open boundary. The ring-shaped lattice may be twisted by introducing an external magnetic field, in which the atoms with nonzero magnetic dipoles experience extra phases. This phase twist cannot be gauged away, leading to persistent currents^60^. Thus, the platform can be used to simulate solid-state Josephson flux qubits^61^, which code the quantum information in the superposition of CW and CCW persistent currents. In addition, attaching the ring-shaped lattice with two leads (source and drain) allows one to study the topological transport^62^.

## Methods

### Magic wavelengths

The general method of calculating the ac polarizability of an atom has been summarized in^37^. The ^88^Sr atoms have zero nuclear spin. In the electric dipole (*E*1) approximation, the ac polarizability of ^88^Sr in $\left| S \right\rangle$ takes the form.

31 $${\alpha }_{\mathrm{S}}\left(\lambda \right)={\sum }_{\mu }\frac{6\pi {\varepsilon }_{0}{c}^{3}{A}_{\mu }}{{\omega }_{\mu }^{2}}\frac{{\omega }_{\mu }^{2}-{\left(2\pi c/\lambda \right)}^{2}+{A}_{\mu }^{2}/4}{{\left({\omega }_{\mu }^{2}-{\left(2\pi c/\lambda \right)}^{2}+{A}_{\mu }^{2}/4\right)}^{2}+{\left(2\pi c/\lambda \right)}^{2}{A}_{\mu }^{2}},$$

where *λ* is the wavelength of an external π-polarization light field, *ω*_*µ*_ is the frequency of the *E*1 transition between the fine-structure upper |*µ*⟩ and lower $\left| S \right\rangle$ states, and *A*_*µ*_ denotes the corresponding Einstein coefficient. The |*µ*⟩ state can be (*nsn*'*p*)^1^*P*_1_ and (5*s*5*p*)^3^*P*_1_. It is seen that ${\alpha }_{\mathrm{S}}\left(\lambda \right)$ is independent of the direction of quantum axis *θ*_B_. By contrast, the ac polarizability of ^88^Sr in $\left| P \right\rangle$ depends on *θ*_B_ and is derived as

32 $$ \alpha _{{\text{P}}} \left( {\lambda ,\theta _{{\text{B}}} } \right) = \sum _{\mu } \left( {\left( {\frac{{{\text{sin}}^{2} \theta _{{\text{B}}} }}{2}\delta _{{J_{\mu } ,1}}  + \frac{{{\text{sin}}^{2} \theta _{{\text{B}}} }}{2}\delta _{{J_{\mu } ,2}}  + \frac{{2{\text{cos}}^{2} \theta _{{\text{B}}} }}{3}\delta _{{J_{\mu } ,2}}  + \frac{{{\text{cos}}^{2} \theta _{{\text{B}}} }}{3}\delta _{{\left| \mu  \right\rangle ,\left( {5p^{2} } \right)^{3} P_{0} }}  - {\text{cos}}^{2} \theta _{{\text{B}}} \delta _{{\left| \mu  \right\rangle ,\left| {\text{S}} \right\rangle }} } \right.} \right)\frac{{6\pi \varepsilon _{0} c^{3} A_{\mu } }}{{\omega _{\mu }^{2} }}\frac{{\omega _{\mu }^{2}  - \left( {2\pi c/\lambda } \right)^{2}  + A_{\mu }^{2} /4}}{{\left( {\omega _{\mu }^{2}  - \left( {2\pi c/\lambda } \right)^{2}  + A_{\mu }^{2} /4} \right)^{2}  + \left( {2\pi c/\lambda } \right)^{2} A_{\mu }^{2} }},$$

where, for example, the value of Kronecker delta ${\delta }_{{J}_{\mu },2}$ is 1 (0) when the total angular moment *J*_*µ*_ of the |*µ*⟩ state is (not) equal to 2. Similarly, ${\delta }_{\left|\mu \right\rangle,\left|\mathrm{S}\right\rangle}=1$ (0) when |*µ*⟩ is (not) $\left| S \right\rangle$. The data of *ω*_*µ*_ and *A*_*µ*_ of the associated *E*1 transitions has been listed in^38^.

**Figure 5:**
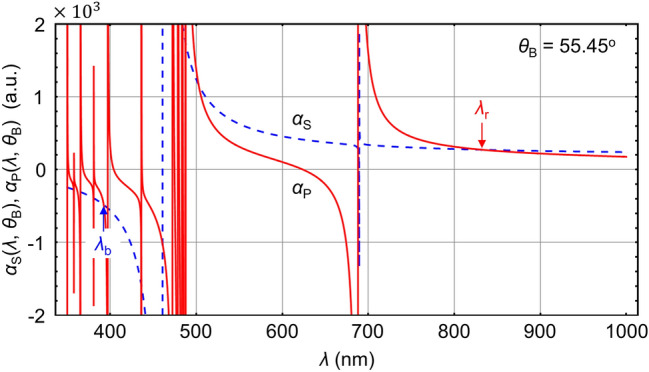
Dependence of dynamic polarizability of ^88^Sr on light wavelength. Polarizabilities ${\alpha }_{j=\mathrm{S},\mathrm{P}}\left(\lambda ,{\theta }_{\text{B}}\right)$ of ^88^Sr in $\left|j=\mathrm{S},\mathrm{P}\right\rangle$ as a function of the trapping-beam wavelength $\lambda$ with ${\theta }_{\text{B}}=55.45^\circ$. The ${\alpha }_{\mathrm{S}}$ curve (dashed) crosses the ${\alpha }_{\mathrm{P}}$ curve (solid) at the red-detuned magic wavelength ${\lambda }_{\mathrm{r}}=832.69$ nm that is resonant to the TE-polarized $\left({n}_{\mathrm{r}}=1,{l}_{\mathrm{r}}=49,\pm {m}_{\mathrm{r}}\right)$ WGMs with ${m}_{\mathrm{r}}=47$. At the blue-detuned wavelength ${\lambda }_{\mathrm{b}}=392.58$ nm, the difference $\mathrm{\Delta }\alpha ={\alpha }_{\mathrm{S}}-{\alpha }_{\mathrm{P}}$ is much smaller than ${\alpha }_{\mathrm{S},\mathrm{P}}$. The wavelength *λ*_b_ is resonant to the TE-polarized $\left({n}_{\mathrm{b}}=1,{l}_{\mathrm{b}}=109,{m}_{\mathrm{b}}=109\right)$ WGM.

Figure 5 illustrates that the difference between ${\alpha }_{\mathrm{S}}$ and ${\alpha }_{\mathrm{P}}$ is cancelled, i.e., ${\alpha }_{\mathrm{r}}\equiv {\alpha }_{\mathrm{S}}\left({\lambda }_{\mathrm{r}}\right)={\alpha }_{\mathrm{P}}\left({\lambda }_{\mathrm{r}}\right)=0.27$ a.u., at ${\lambda }_{\mathrm{r}}=832.69$ nm with ${\theta }_{\text{B}}=55.45^\circ$. The magic wavelength *λ*_r_ is red-detuned to the intercombination transition of ^88^Sr and matches the $\left({n}_{\mathrm{r}}=1,{l}_{\mathrm{r}}=49,\pm {m}_{\mathrm{r}}\right)$ WGMs with ${m}_{\mathrm{r}}=47$. Generally, it is challenging to find the other magic wavelength that is blue-detuned and also coincides with a WGM of microsphere. Nevertheless, the wavelength of the blue-detuned trapping beam can be chosen as ${\lambda }_{\mathrm{b}}=392.58$ nm, which is resonant to the fundamental $\left({n}_{\mathrm{b}}=1,{l}_{\mathrm{b}}=109,{m}_{\mathrm{b}}=109\right)$ WGM and is close to the other blue-detuned magic wavelength at 392.44 nm. At *λ*_b_, we have ${\alpha }_{\mathrm{S}}\left({\lambda }_{\mathrm{b}},{\theta }_{\text{B}}\right)=-0.48$ a.u. and ${\alpha }_{\mathrm{P}}\left({\lambda }_{\mathrm{b}},{\theta }_{\text{B}}\right)=-0.49$ a.u. (Fig. 5). Since the relative difference $\left({\alpha }_{\mathrm{S}}-{\alpha }_{\mathrm{P}}\right)/{\alpha }_{\mathrm{S},\mathrm{P}}$ is about $-2.1\times {10}^{-2}$, one may approximate ${\alpha }_{\mathrm{b}}\equiv {\alpha }_{\mathrm{S}}\left({\lambda }_{\mathrm{b}},{\theta }_{\text{B}}\right)\approx {\alpha }_{\mathrm{P}}\left({\lambda }_{\mathrm{b}},{\theta }_{\text{B}}\right)$ at *λ*_b_.

The atoms scattering the photons of the trapping beams heats the temperature of atoms. The lifetime of confined atoms in $\left| S \right\rangle$ is determined by the reciprocal of the photon scattering rate.

33 $${\gamma }_{\text{p}}={\sum }_{i=\mathrm{r},\mathrm{b}}{\sum }_{\mu }\frac{{I}_{i}/{I}_{\mathrm{s}\mathrm{a}\mathrm{t},\mu }}{1+{I}_{i}/{I}_{\mathrm{s}\mathrm{a}\mathrm{t},\mu }+4{\left({\omega }_{i}-{\omega }_{\mu }\right)}^{2}/{A}_{\mu }^{2}}{A}_{\mu },$$

where ${I}_{i}$ denotes the red/blue-detuned light intensity at the location of the atom, ${\omega }_{i}=2\mathrm{\pi }c/{\lambda }_{i}$ is the trapping beam frequency, and ${I}_{\mathrm{s}\mathrm{a}\mathrm{t},\mu }=\left(2\mathrm{\pi }hc/3{\lambda }_{\mu }^{3}\right){A}_{\mu }$ gives the saturation intensity corresponding to the |*µ*⟩ – $\left| S \right\rangle$ transition with ${\lambda }_{\mu }=2\mathrm{\pi }c/{\omega }_{\mu }$. We set the light powers of the red-detuned and blue-detuned trapping beams in the $y-z$ plane at 27 and 87 mW, respectively. The atoms are confined at a location with a distance of $\left({\rho }_{0}-R\right)=137$ nm to the microsphere’s surface. The resulting *γ*_p_ is ~ 10^−3^ s^−1^ and the trapping lifetime is estimated to be 10^3^ s. By contrast, the lifetime of the confined atoms in $\left| P \right\rangle$ is primarily determined by the intrinsic decay rate *γ* of the intercombination transition.

### Vibrational states of an atom in a lattice site

In spherical coordinates $\left(\rho >R,\theta ,\varphi \right)$, the blue- and red-detuned optical potentials have the approximate expressions.

34a $${U}_{\mathrm{b}}\left(\mathbf{r}\right)=-\left({\alpha }_{\mathrm{b}}/4\right){z}_{{l}_{\mathrm{b}}}^{2}\left(\rho \right){\mathrm{\Theta }}_{{l}_{\mathrm{b}},{l}_{\mathrm{b}}}^{2}\left(\theta \right),$$

34b $${U}_{\mathrm{r}}\left(\mathbf{r}\right)=-{\alpha }_{\mathrm{r}}{z}_{{l}_{\mathrm{r}}}^{2}\left(\rho \right){\mathrm{\Theta }}_{{l}_{\mathrm{r}},{m}_{\mathrm{r}}}^{2}\left(\theta \right){\mathrm{cos}}^{2}{m}_{\mathrm{r}}\varphi ,$$

with ${z}_{{l}_{u=\mathrm{b},\mathrm{r}}}\left(\rho \right)={\xi }_{{l}_{u}}\left({k}_{u}\rho \right)/{k}_{u}\rho$. Adding van der Waals potential, the total lattice potential is expressed as ${U}_{\mathrm{t}\mathrm{o}\mathrm{t}}\left(\mathbf{r}\right)={U}_{\mathrm{b}}\left(\mathbf{r}\right)+{U}_{\mathrm{r}}\left(\mathbf{r}\right)-{C}_{3}/{\left(\rho -R\right)}^{3}$. We assume that the lattice potential is deep enough that the atoms are tightly confined within the central region of each lattice site. Let us focus on the lattice site located at $\left({\rho }_{0},\theta =\mathrm{\pi }/2,\varphi =0\right)$. Writing $\theta =\mathrm{\pi }/2+\delta \theta /\sqrt{{l}_{\mathrm{b}}}$ and $\varphi =\delta \varphi /{m}_{\mathrm{r}}$ with small angles $\delta \theta$ and $\delta \varphi$, one has the approximations ${\mathrm{\Theta }}_{{l}_{\mathrm{b}},{l}_{\mathrm{b}}}^{2}\left(\theta \right)\approx \sqrt{{l}_{\mathrm{b}}/\pi }\left[1-{\left(\delta \theta \right)}^{2}\right]$, ${\mathrm{cos}}^{2}{m}_{\mathrm{r}}\varphi \approx \left[1-{\left(\delta \varphi \right)}^{2}\right]$, and ${\mathrm{\Theta }}_{{l}_{\mathrm{r}},{m}_{\mathrm{r}}}^{2}\left(\theta \right)\approx \sqrt{{m}_{\mathrm{r}}/64\pi }\left[1-\left(3{m}_{\mathrm{r}}/{l}_{\mathrm{b}}\right){\left(\delta \theta \right)}^{2}\right]$. Thus, ${U}_{\mathrm{t}\mathrm{o}\mathrm{t}}\left(\mathbf{r}\right)$ is rewritten as

$${U}_{\mathrm{t}\mathrm{o}\mathrm{t}}\left(\mathbf{r}\right)\approx \left[-\frac{{\alpha }_{\mathrm{b}}}{4}{z}_{{l}_{\mathrm{b}}}^{2}\left(\rho \right)\sqrt{\frac{{l}_{\mathrm{b}}}{\mathrm{\pi }}}-{\alpha }_{\mathrm{r}}{z}_{{l}_{\mathrm{r}}}^{2}\left(\rho \right)\sqrt{\frac{{m}_{\mathrm{r}}}{64\mathrm{\pi }}}-\frac{{C}_{3}}{{\left(\rho -R\right)}^{3}}\right]$$

$$+\left[\frac{{\alpha }_{\mathrm{b}}}{4}{z}_{{l}_{\mathrm{b}}}^{2}\left(\rho \right)\sqrt{\frac{{l}_{\mathrm{b}}}{\mathrm{\pi }}}+{\alpha }_{\mathrm{r}}{z}_{{l}_{\mathrm{r}}}^{2}\left(\rho \right)\frac{3{m}_{\mathrm{r}}}{{l}_{\mathrm{b}}}\sqrt{\frac{{m}_{\mathrm{r}}}{64\mathrm{\pi }}}\right]{\left(\delta \theta \right)}^{2}$$

35 $$+\left[{\alpha }_{\mathrm{r}}{z}_{{l}_{\mathrm{r}}}^{2}\left(\rho \right)\sqrt{\frac{{m}_{\mathrm{r}}}{64\mathrm{\pi }}}\right]{\left(\delta \varphi \right)}^{2}.$$

The Hamiltonian for an atom (mass *M*) moving in ${U}_{\mathrm{t}\mathrm{o}\mathrm{t}}\left(\mathbf{r}\right)$ takes the form.

36 $$H=-\frac{{\mathrm{\hslash }}^{2}}{2M}\left(\frac{{\partial }^{2}}{\partial {\rho }^{2}}+\frac{2}{\rho }\frac{\partial }{\partial \rho }+\frac{1}{{\rho }^{2}\mathrm{sin}\theta }\frac{\partial }{\partial \theta }\mathrm{sin}\theta \frac{\partial }{\partial \theta }+\frac{1}{{\rho }^{2}{\mathrm{sin}}^{2}\theta }\frac{{\partial }^{2}}{\partial {\varphi }^{2}}\right)+{U}_{\mathrm{t}\mathrm{o}\mathrm{t}}\left(\mathbf{r}\right).$$

Using $\frac{1}{\mathrm{sin}\theta }\frac{\partial }{\partial \theta }\mathrm{sin}\theta \frac{\partial }{\partial \theta }\sim {l}_{\mathrm{b}}\frac{{\partial }^{2}}{\partial {\left(\delta \theta \right)}^{2}}$ and $\frac{1}{{\mathrm{sin}}^{2}\theta }\sim 1$, *H* is approximately re-expressed as

$$H\approx \left[-\frac{{\hslash }^{2}}{2M}\left(\frac{{\partial }^{2}}{\partial {\rho }^{2}}+\frac{2}{\rho }\frac{\partial }{\partial \rho }\right)+{U}_{\rho }\left(\rho \right)\right]+\left[-\frac{{\hslash }^{2}}{2M}{l}_{\mathrm{b}}\frac{{\partial }^{2}}{\partial {\left({\rho }_{0}\delta \theta \right)}^{2}}+{U}_{\theta }\left({\rho }_{0}\right){\left(\delta \theta \right)}^{2}\right]$$

37 $$+\left[-\frac{{\mathrm{\hslash }}^{2}}{2M}{m}_{\mathrm{r}}^{2}\frac{{\partial }^{2}}{\partial {\left({\rho }_{0}\delta \varphi \right)}^{2}}+{U}_{\varphi }\left({\rho }_{0}\right){\left(\delta \varphi \right)}^{2}\right],$$

where we have defined three potential components.

38a $${U}_{\rho }\left(\rho \right)=-\frac{{\alpha }_{\mathrm{b}}}{4}{z}_{{l}_{\mathrm{b}}}^{2}\left(\rho \right)\sqrt{\frac{{l}_{\mathrm{b}}}{\mathrm{\pi }}}-{\alpha }_{\mathrm{r}}{z}_{{l}_{\mathrm{r}}}^{2}\left(\rho \right)\sqrt{\frac{{m}_{\mathrm{r}}}{64\mathrm{\pi }}}-\frac{{C}_{3}}{{\left(\rho -R\right)}^{3}},$$

38b $${U}_{\theta }\left({\rho }_{0}\right)=\frac{{\alpha }_{\mathrm{b}}}{4}{z}_{{l}_{\mathrm{b}}}^{2}\left({\rho }_{0}\right)\sqrt{\frac{{l}_{\mathrm{b}}}{\mathrm{\pi }}}+{\alpha }_{\mathrm{r}}{z}_{{l}_{\mathrm{r}}}^{2}\left({\rho }_{0}\right)\sqrt{\frac{{m}_{\mathrm{r}}}{64\mathrm{\pi }}}\frac{3{m}_{\mathrm{r}}}{{l}_{\mathrm{b}}},$$

38c $${U}_{\varphi }\left({\rho }_{0}\right)={\alpha }_{\mathrm{r}}{z}_{{l}_{\mathrm{r}}}^{2}\left({\rho }_{0}\right)\sqrt{\frac{{m}_{\mathrm{r}}}{64\mathrm{\pi }}.}$$

**Figure 6:**
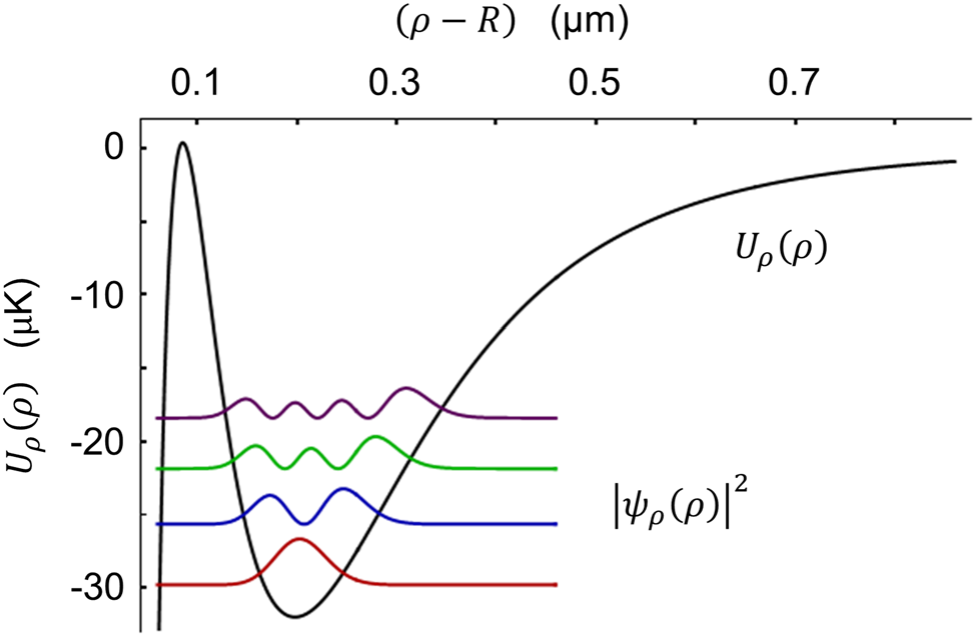
Vibrational states in radial potential. Several lowest vibrational states ${\psi }_{\rho }\left(\rho \right)$ of an atom moving inside the potential ${U}_{\rho }\left(\rho \right)$ along the radial direction. The potential depth is 32 K$\mu$. The vibrational states are separated with an approximately equal spacing of ${\mathrm{\Omega }}_{\rho }=2\mathrm{\pi }\times 86$ kHz. The corresponding Lamb–Dicke parameter is evaluated to be ${\eta }_{\rho }=0.23$.

Following the approach of separation of variables, the Schrödinger equation $H\mathrm{\Psi }\left(\text{r}\right)=E\mathrm{\Psi }\left(\text{r}\right)$ leads to.

39a $$\left[-\frac{{\mathrm{\hslash }}^{2}}{2M}\left(\frac{{\partial }^{2}}{\partial {\rho }^{2}}+\frac{2}{\rho }\frac{\partial }{\partial \rho }\right)+{U}_{\rho }\left(\rho \right)\right]{\psi }_{\rho }\left(\rho \right)={E}_{\rho }{\psi }_{\rho }\left(\rho \right),$$

39b $$\left[-\frac{{\mathrm{\hslash }}^{2}}{2M}{m}_{\mathrm{r}}^{2}\frac{{\partial }^{2}}{\partial {\left({\rho }_{0}\delta \varphi \right)}^{2}}+{U}_{\varphi }\left({\rho }_{0}\right){\left(\delta \varphi \right)}^{2}\right]{\psi }_{\varphi }\left(\delta \varphi \right)={E}_{\varphi }{\psi }_{\varphi }\left(\delta \varphi \right),$$

39c $$\left[-\frac{{\mathrm{\hslash }}^{2}}{2M}{l}_{\mathrm{b}}\frac{{\partial }^{2}}{\partial {\left({\rho }_{0}\delta \theta \right)}^{2}}+{U}_{\theta }\left({\rho }_{0}\right){\left(\delta \theta \right)}^{2}\right]{\psi }_{\theta }\left(\delta \theta \right)={E}_{\theta }{\psi }_{\theta }\left(\delta \theta \right),$$

with the egeinfunction $\mathrm{\Psi }\left(\rho ,\theta ,\varphi \right)={\psi }_{\rho }\left(\rho \right){\psi }_{\theta }\left(\delta \theta \right){\psi }_{\varphi }\left(\delta \varphi \right)$ and the eigenvalue $E={E}_{\rho }+{E}_{\theta }+{E}_{\varphi }$. The radial wavefunction ${\psi }_{\rho }\left(\rho \right)$ can be solved in a numerical way. We consider ${U}_{\mathrm{t}\mathrm{o}\mathrm{t}}\left(\mathbf{r}\right)$ shown in Fig. 2b. Figure 6 depicts the distributions ${\left|{\psi }_{\rho }\left(\rho \right)\right|}^{2}$ of the first few eigenstates with a nearly equal energy separation of ${\mathrm{\Omega }}_{\rho }=2\mathrm{\pi }\times 86$ kHz. By contrast, the atom acts as a quantum harmonic oscillator along either $\theta$- or $\varphi$-direction with the corresponding oscillation frequencies ${\mathrm{\Omega }}_{\theta }=2\mathrm{\pi }\times 25$ kHz and ${\mathrm{\Omega }}_{\varphi }=2\mathrm{\pi }\times 145$ kHz, respectively.

## Data Availability

All data supporting the findings of this study are available from the corresponding author upon reasonable request.

## Code availability

The computer code to simulate the dynamics are available from the corresponding author upon reasonable request.

## References

[1] Zhuang W, Yu D, Liu Z, Chen J (2011). Multi-threshold second-order phase transition in laser. Chi. Sci. Bull..

[2] De Martini F, Cairo F, Mataloni P, Verzegnassi F (1992). Thresholdless microlaser. Phys. Rev. A.

[3] McKeever J, Boca A, Boozer AD, Buck JR, Kimble HJ (2003). Experimental realization of a one-atom laser in the regime of strong coupling. Nature.

[4] Zhou Z, Yin B, Michel J (2015). On-chip light sources for silicon photonics. Light Sci. Appl..

[5] Oulton RF (2009). Plasmon lasers at deep subwavelength scale. Nature.

[6] Kok P, Munro WJ, Nemoto K, Ralph TC, Dowling JP, Milburn GJ (2007). Linear optical quantum computing with photonic qubits. Rev. Mod. Phys..

[7] Li B-B (2014). Single nanoparticle detection using split-mode microcavity Raman lasers. PNAS.

[8] Toropov N (2021). Review of biosensing with whispering-gallery mode lasers. Light Sci. Appl..

[9] Miao P (2016). Orbital angular momentum microlaser. Science.

[10] Kimble HJ (1998). Strong interactions of single atoms and photons in cavity QED. Phys. Scr..

[11] Takahashi H, Kassa E, Christoforou C, Keller M (2020). Strong coupling of a single ion to an optical cavity. Phys. Rev. Lett..

[12] Wang D (2019). Turning a molecule into a coherent two-level quantum system. Nat. Phys..

[13] Nomura M, Kumagai N, Iwamoto S, Ota Y, Arakawa Y (2010). Laser oscillation in a strongly coupled single-quantum-dot-nanocavity system. Nat. Phys..

[14] Wallraff A (2004). Strong coupling of a single photon to a superconducting qubit using circuit quantum electrodynamics. Nature.

[15] Chikkaraddy R (2016). Single-molecule strong coupling at room temperature in plasmonic nanocavities. Nature.

[16] Gorodetsky ML, Savchenkov AA, Ilchenko VS (1996). Ultimate Q of optical microsphere resonators. Opt. Lett..

[17] Vernooy DW, Ilchenko VS, Mabuchi H, Streed EW, Kimble HJ (1998). High-Q measurements of fused-silica microspheres in the near infrared. Opt. Lett..

[18] Balac S (2019). WGMode: A Matlab toolbox for whispering gallery modes volume computation in spherical optical micro-resonators. Comput. Phys. Commun..

[19] Buck JR, Kimble HJ (2003). Optimal sizes of dielectric microspheres for cavity QED with strong coupling. Phys. Rev. A.

[20] Spillane SM (2005). Ultrahigh- toroidal microresonators for cavity quantum electrodynamics. Phys. Rev. A.

[21] Aoki T (2006). Observation of strong coupling between one atom and a monolithic microresonator. Nature.

[22] Alton DJ (2011). Strong interactions of single atoms and photons near a dielectric boundary. Nat. Phys..

[23] Bohnet JG, Chen Z, Weiner JM, Cox KC, Thompson JK (2013). Active and passive sensing of collective atomic coherence in a superradiant laser. Phys. Rev. A.

[24] Norcia MA, Winchester MN, Cline JRK, Thompson JK (2016). Superradiance on the millihertz linewidth strontium clock transition. Sci. Adv..

[25] Yoon S (2006). Definitive number of atoms on demand: Controlling the number of atoms in a few-atom magneto-optical trap. Appl. Phys. Lett..

[26] Ido T, Katori H (2003). Recoil-free spectroscopy of neutral Sr atoms in the Lamb-Dicke regime. Phys. Rev. Lett..

[27] Katori, H., Takamoto, M., Pal’chikov, V. G. & Ovsiannikov, V. D. Ultrastable optical clock with neutral atoms in an engineered light shift trap. *Phys. Rev. Lett.* **91**, 173005 (2003). 10.1103/PhysRevLett.91.173005 14611343

[28] Mabuchi H, Kimble HJ (1994). Atom galleries for whispering atoms: binding atoms in stable orbits around an optical resonator. Opt. Lett..

[29] Vernooy DW, Kimble HJ (1997). Quantum structure and dynamics for atom galleries. Phys. Rev. A.

[30] Rosenblit M, Japha Y, Horak P, Folman R (2006). Simultaneous optical trapping and detection of atoms by microdisk resonators. Phys. Rev. A.

[31] Kien FL, Balykin VI, Hakuta K (2004). Atom trap and waveguide using a two-color evanescent light field around a subwavelength-diameter optical fiber. Phys. Rev. A.

[32] Vetsch E (2010). Optical interface created by laser-cooled atoms trapped in the evanescent field surrounding an optical nanofiber. Phys. Rev. Lett..

[33] Lacroûte C (2012). A state-insensitive, compensated nanofiber trap. New J. Phys..

[34] Goban A (2012). Demonstration of a state-insensitive, compensated nanofiber trap. Phys. Rev. Lett..

[35] Lam CC, Leung PT, Young K (1992). Explicit asymptotic formulas for the positions, widths, and strengths of resonances in Mie scattering. J. Opt. Soc. Am. B.

[36] Vollmer F, Yu D (2020). Optical Whispering Gallery Modes for Biosensing: From Physical Principles to Applications.

[37] Rosenbusch P (2009). ac Stark shift of the Cs microwave atomic clock transitions. Phys. Rev. A.

[38] Zhou X, Xu X, Chen X, Chen J (2010). Magic wavelengths for terahertz clock transitions. Phys. Rev. A.

[39] Takamoto M, Katori H, Marmo SI, Ovsiannikov VD, Palchikov VG (2009). Prospects for optical clocks with a blue-detuned lattice. Phys. Rev. Lett..

[40] Katori H, Ido T, Isoya Y, Kuwata-Gonokami M (1999). Magneto-optical trapping and cooling of strontium atoms down to the photon recoil temperature. Phys. Rev. Lett..

[41] Savchenkov AA, Matsko AB, Ilchenko VS, Strekalov D, Maleki L (2007). Direct observation of stopped light in a whispering-gallery-mode microresonator. Phys. Rev. A.

[42] Carmon T (2008). Static envelope patterns in composite resonances generated by level crossing in optical toroidal microcavities. Phys. Rev. Lett..

[43] Attar ST, Shuvayev V, Deych L, Martin LL, Carmon T (2016). Level-crossing and modal structure in microdroplet resonators. Opt. Express.

[44] Meiser D, Ye J, Carlson DR, Holland MJ (2009). Prospects for a millihertz-linewidth laser. Phys. Rev. Lett..

[45] Spillane, S. M. *Fiber-coupled ultra-high- microresonators for nonlinear and quantum optics.* Dissertation (Ph.D.), California Institute of Technology (2004).

[46] Blanc W, Dussardier B (2016). Formation and applications of nanoparticles in silica optical fibers. J. Opt..

[47] Hale GM, Querry MR (1973). Optical constants of water in the 200-nm to 200-m wavelength region. Appl. Opt..

[48] Yu D (2016). Two coupled one-atom lasers. J. Opt. Soc. Am. B.

[49] Mølmer K, Castin Y, Dalibard J (1993). Monte Carlo wave-function method in quantum optics. J. Opt. Soc. Am. B.

[50] Purcell EM, Torrey HC, Pound RV (1946). Resonance absorption by nuclear magnetic moments in a solid. Phys. Rev..

[51] Hadfield RH (2009). Single-photon detectors for optical quantum information applications. Nat. Photon..

[52] Hanbury Brown R, Twiss RQ (1956). Correlation between photons in two coherent beams of light. Nature.

[53] Macovei M, Evers J, Keitel CH (2005). Quantum correlations of an atomic ensemble via an incoherent bath. Phys. Rev. A.

[54] Löffler M, Meyer GM, Walther H (1997). Spectral properties of the one-atom laser. Phys. Rev. A.

[55] Harris SE (1989). Lasers without inversion: Interference of lifetime-broadened resonances. Phys. Rev. Lett..

[56] Zibrov AS (1995). Experimental demonstration of laser oscillation without population inversion via quantum interference in Rb. Phys. Rev. Lett..

[57] Maier T, Kraemer S, Ostermann L, Ritsch HA (2014). Superradiant clock laser on a magic wavelength optical lattice. Opt. Express.

[58] Chen J (2009). Active optical clock. Chin. Sci. Bull..

[59] Campbell SL (2017). A Fermi-degenerate three-dimensional optical lattice clock. Science.

[60] Amico L, Osterloh A, Cataliotti F (2005). Quantum many particle systems in ring-shaped optical lattices. Phys. Rev. Lett..

[61] Amico L (2014). Superfluid qubit systems with ring shaped optical lattices. Sci. Rep..

[62] Haug T, Dumke R, Kwek L-C, Amico L (2019). Topological pumping in Aharonov-Bohm rings. Commun. Phys..

